# Supplementary material for: Development of Novel Peptidyl Nitriles Targeting Rhodesain and Falcipain-2 for the Treatment of Sleeping Sickness and Malaria
Source: Int J Mol Sci. 2024 Apr 17;25(8):4410. doi: 10.3390/ijms25084410 (PMC11050014; doi:10.3390/ijms25084410)
Supplement: Supplementary file 1 [file ijms-25-04410-s001.zip › ijms-2953199-supplementary.pdf]

Supplementary material

Figure captions

Figure S1. ^{1}H NMR spectrum of compound **14a**.

Figure S2. ^{1}H NMR spectrum of compound **15a**.

Figure S3. ^{1}H NMR spectrum of compound **16a.**

Figure S4. ^{1}H NMR spectrum of compound **17a.**

Figure S5. ^{1}H NMR spectrum of compound **14b.**

Figure S6. ^{1}H NMR spectrum of compound **15b**.

Figure S7. ^{1}H NMR spectrum of compound **16b.**

Figure S8. ^{1}H NMR spectrum of compound **17b**.

Figure S9. ^{1}H NMR spectrum of compound **18a.**

Figure S10. ^{1}H NMR spectrum of compound **19a**.

Figure S11. ^{1}H NMR spectrum of compound **20a.**

Figure S12. ^{1}H NMR spectrum of compound **21a**.

Figure S13. ^{1}H NMR spectrum of compound **18b.**

Figure S14. ^{1}H NMR spectrum of compound 1**9b.**

Figure S15. ^{1}H NMR spectrum of compound **20b.**

Figure S16. ^{1}H NMR spectrum of compound **21b.**

Figure S17. ^{1}H NMR spectrum of compound **1a.**

Figure S18. ^{13}C NMR spectrum of compound **1a.**

Figure S19. ^{1}H NMR spectrum of compound **2a.**

Figure S20. ^{13}C NMR spectrum of compound **2a**.

Figure S21. ^{1}H NMR spectrum of compound **3a.**

Figure S22. ^{13}C NMR spectrum of compound **3a**.

Figure S23. ^{1}H NMR spectrum of compound **4a.**

Figure S24. ^{13}C NMR spectrum of compound **4a**.

Figure S25. ^{1}H NMR spectrum of compound **1b.**

Figure S26. ^{13}C NMR spectrum of compound **1b**.

Figure S27. ^{1}H NMR spectrum of compound **2b.**

Figure S28. ^{13}C NMR spectrum of compound **2b**.

Figure S29. ^{1}H NMR spectrum of compound **3b.**

Figure S30. ^{13}C NMR spectrum of compound **3b**.

Figure S31. ^{1}H NMR spectrum of compound **4b.**

Figure S32. ^{13}C NMR spectrum of compound **4b**.

Figure S33. ^{1}H NMR spectrum of compound **5a.**

Figure S34. ^{13}C NMR spectrum of compound **5a**.

Figure S35. ^{1}H NMR spectrum of compound **6a.**

Figure S36. ^{13}C NMR spectrum of compound **6a**.

Figure S37. ^{1}H NMR spectrum of compound **7a.**

Figure S38. ^{13}C NMR spectrum of compound 7**a**.

Figure S39. ^{1}H NMR spectrum of compound **8a.**

Figure S40. ^{13}C NMR spectrum of compound **8a**.

Figure S41. ^{1}H NMR spectrum of compound **5b**.

Figure S42. ^{13}C NMR spectrum of compound **5b.**

Figure S43. ^{1}H NMR spectrum of compound **6b**.

Figure S44. ^{13}C NMR spectrum of compound **6b**.

Figure S45. ^{1}H NMR spectrum of compound **7b.**

Figure S46. ^{13}C NMR spectrum of compound **7b**.

Figure S47. ^{1}H NMR spectrum of compound **8b.**

Figure S48. ^{13}C NMR spectrum of compound **8b**.

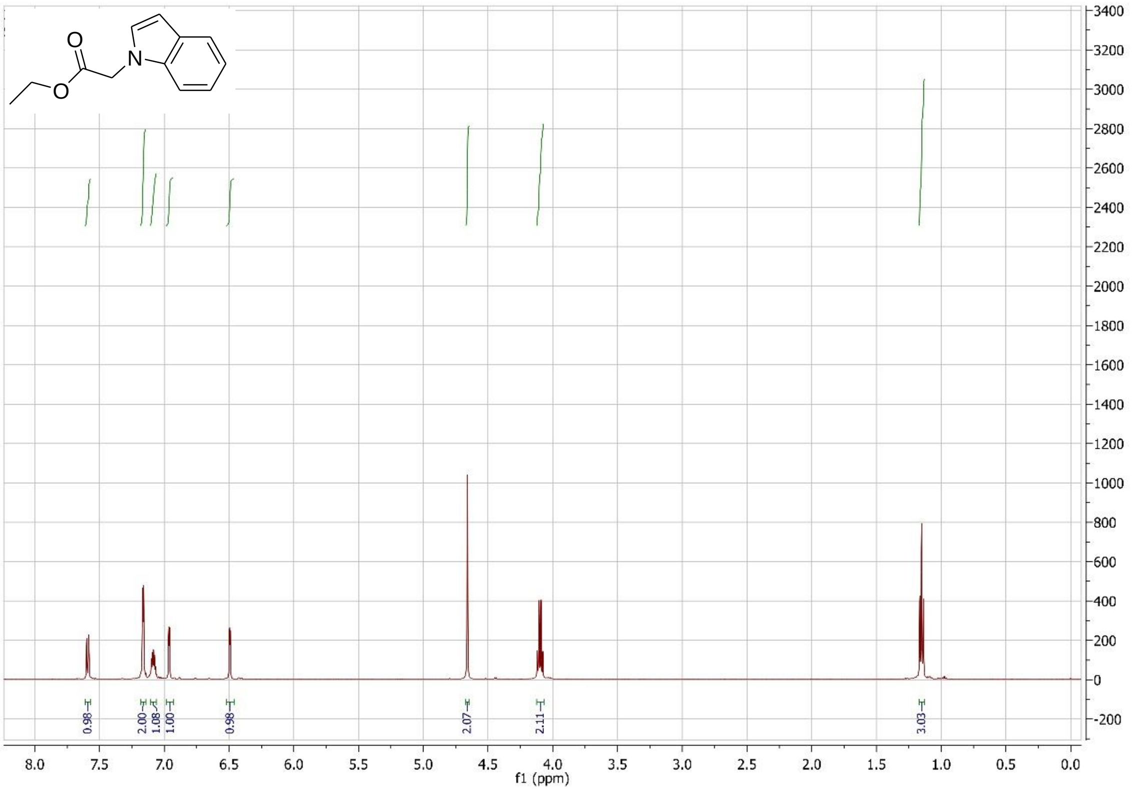

Figure S1. ^{1}H NMR spectrum of compound **14a**.

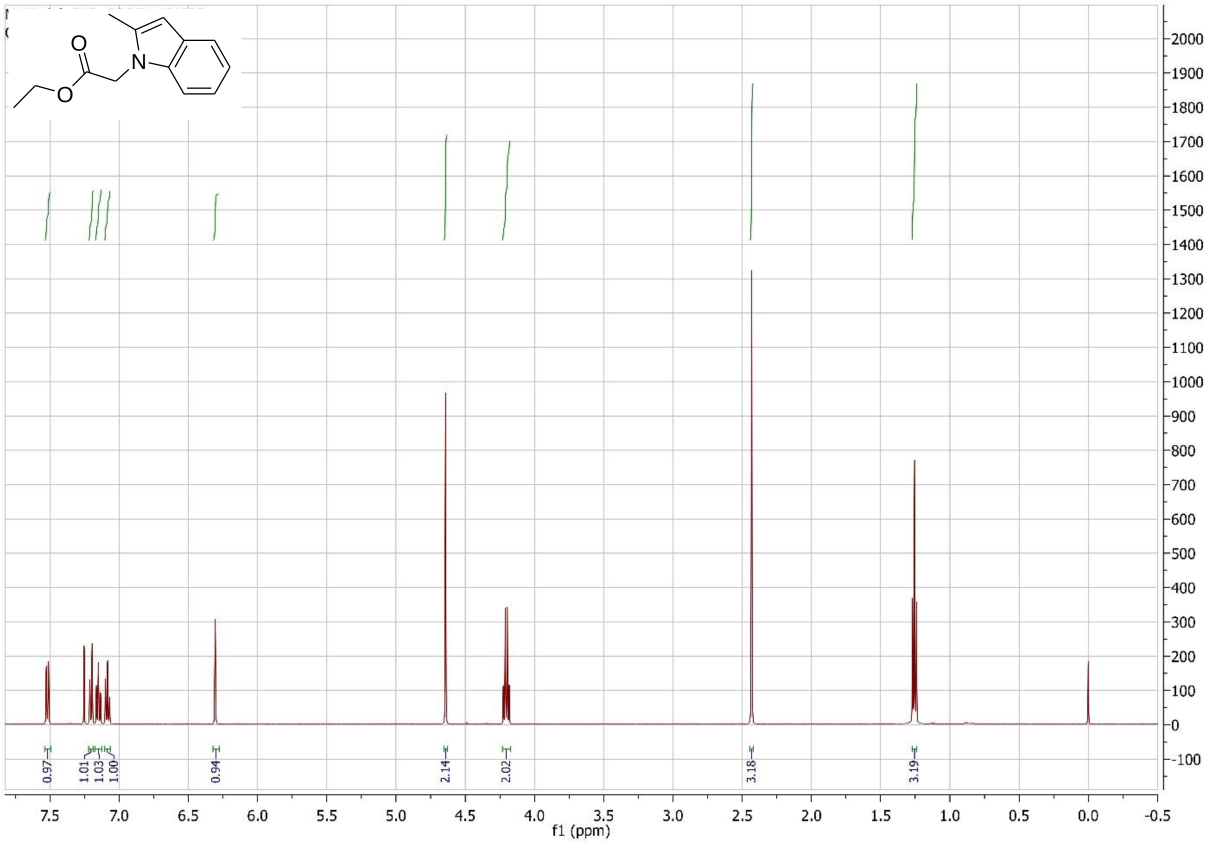

Figure S2. ^{1}H NMR spectrum of compound **15a.**

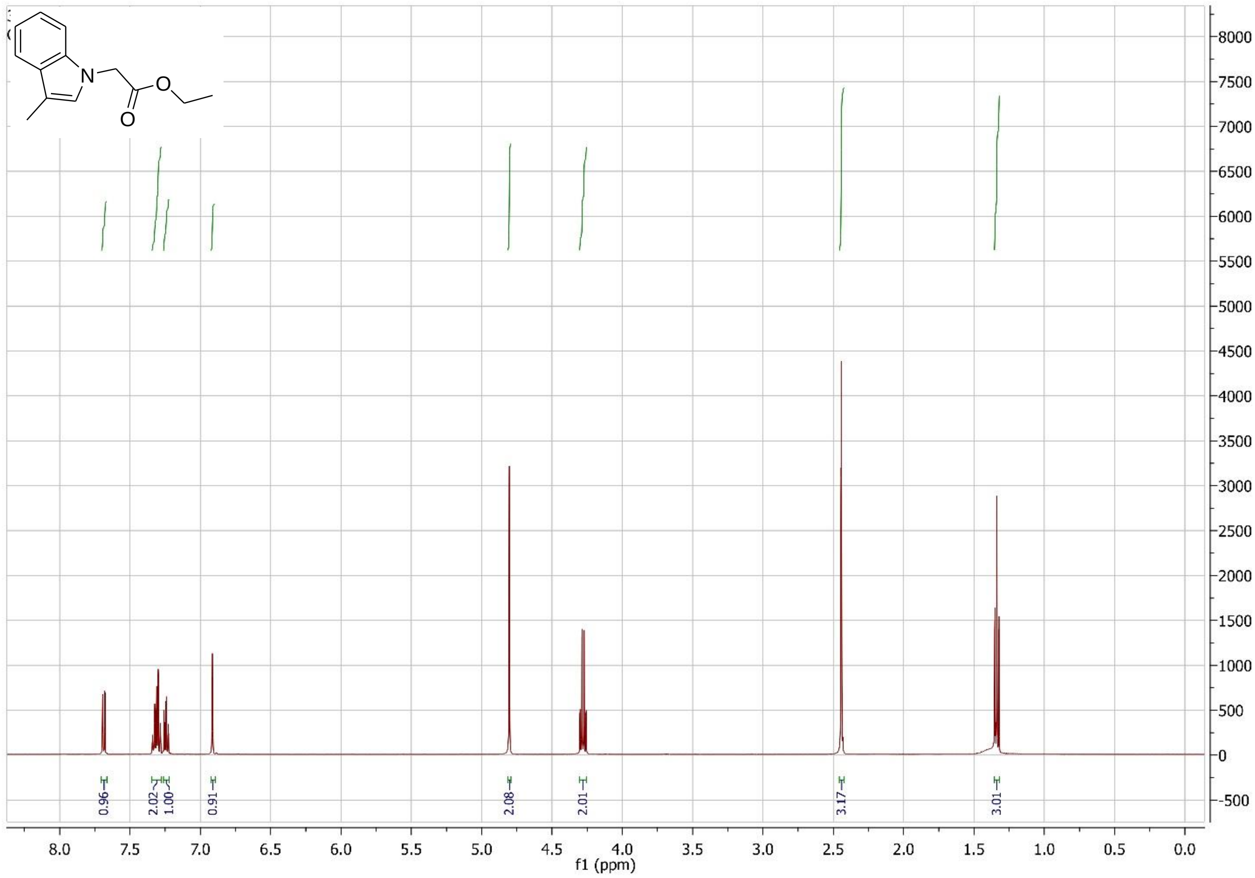

Figure S3. ^{1}H NMR spectrum of compound **16a.**

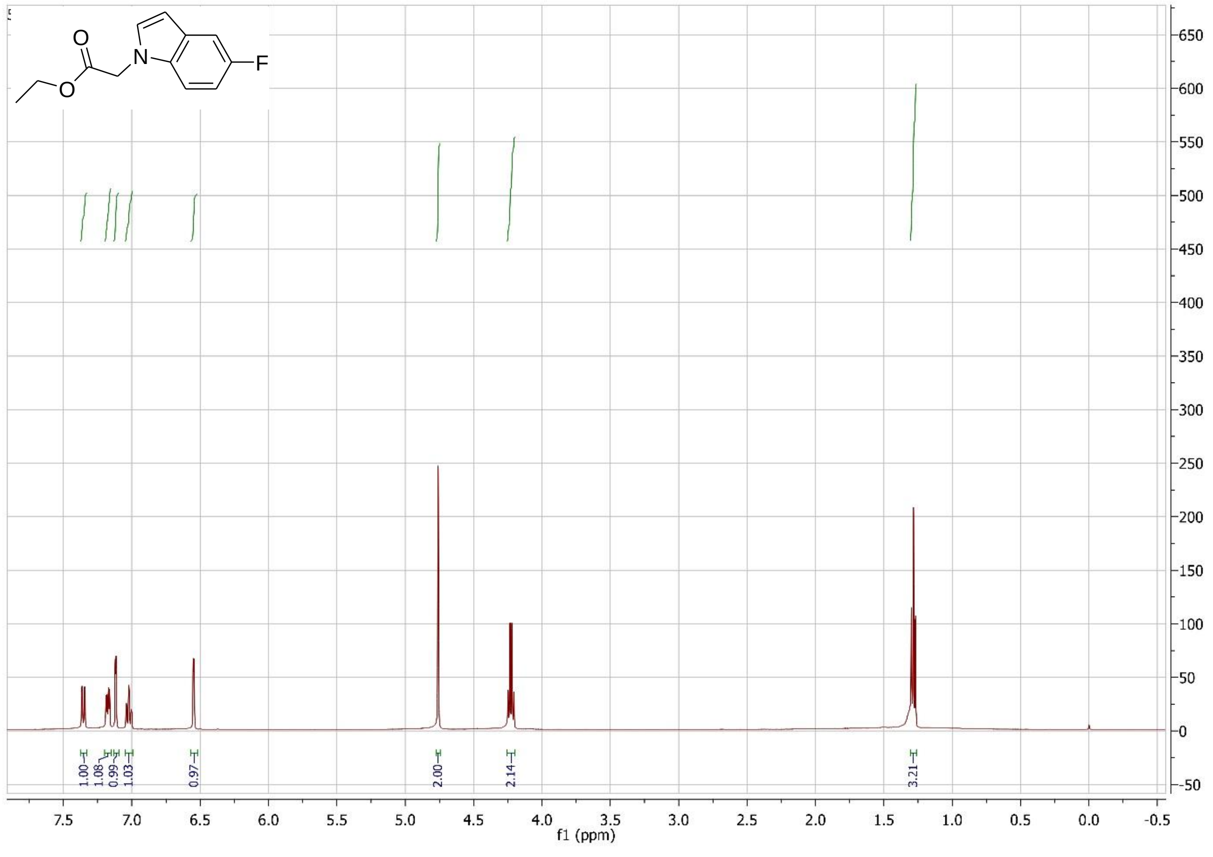

Figure S4. ^{1}H NMR spectrum of compound **17a.**

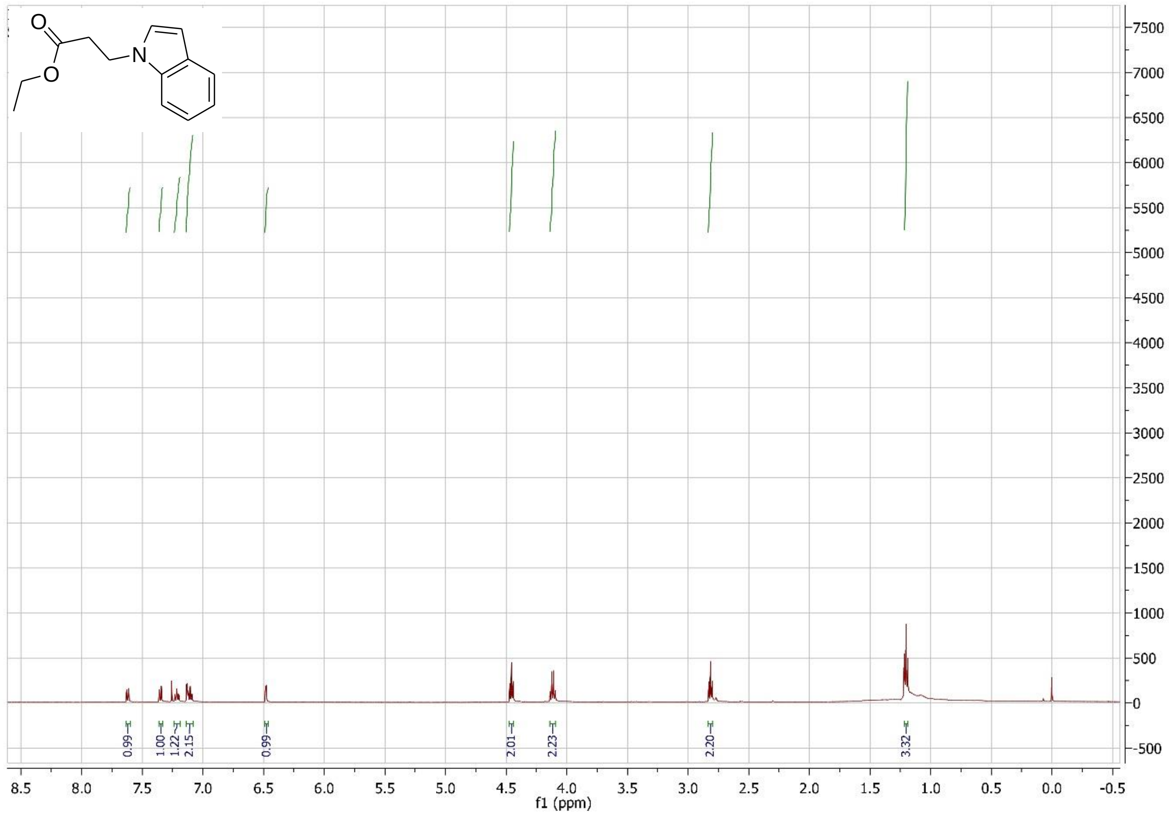

Figure S5. ^{1}H NMR spectrum of compound **14b.**

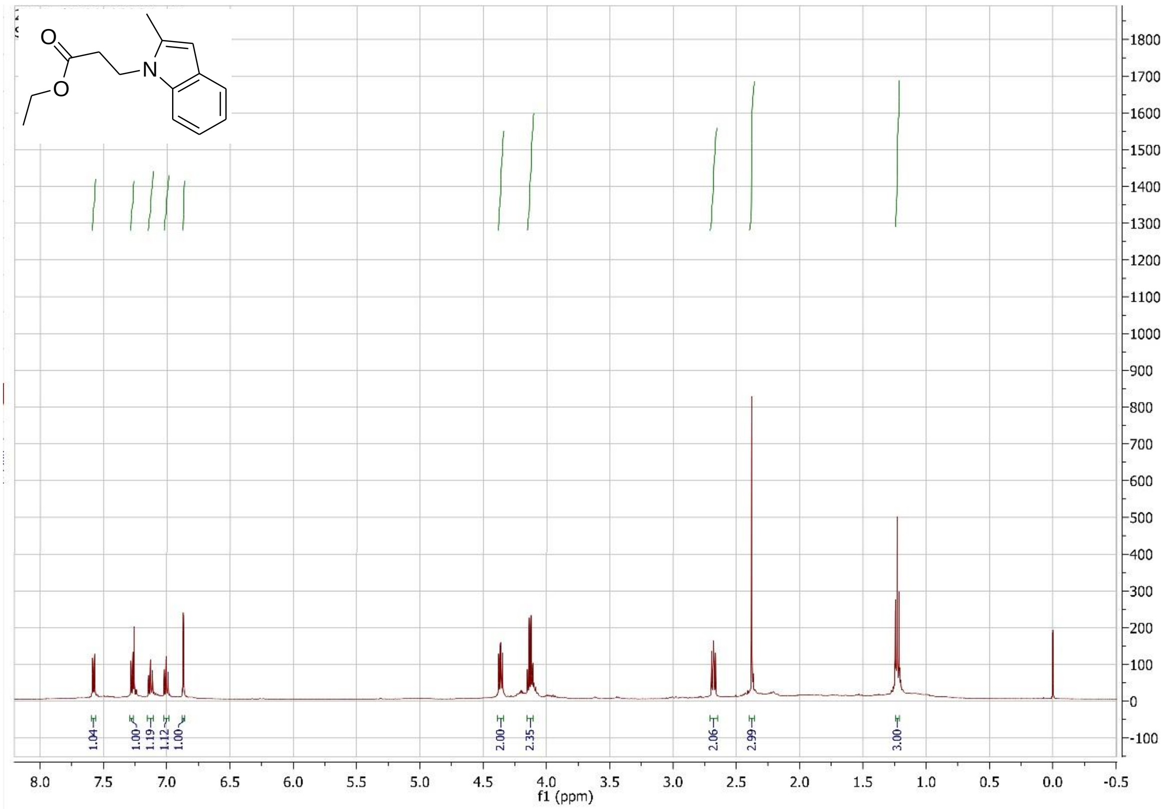

Figure S6. ^{1}H NMR spectrum of compound **15b**.

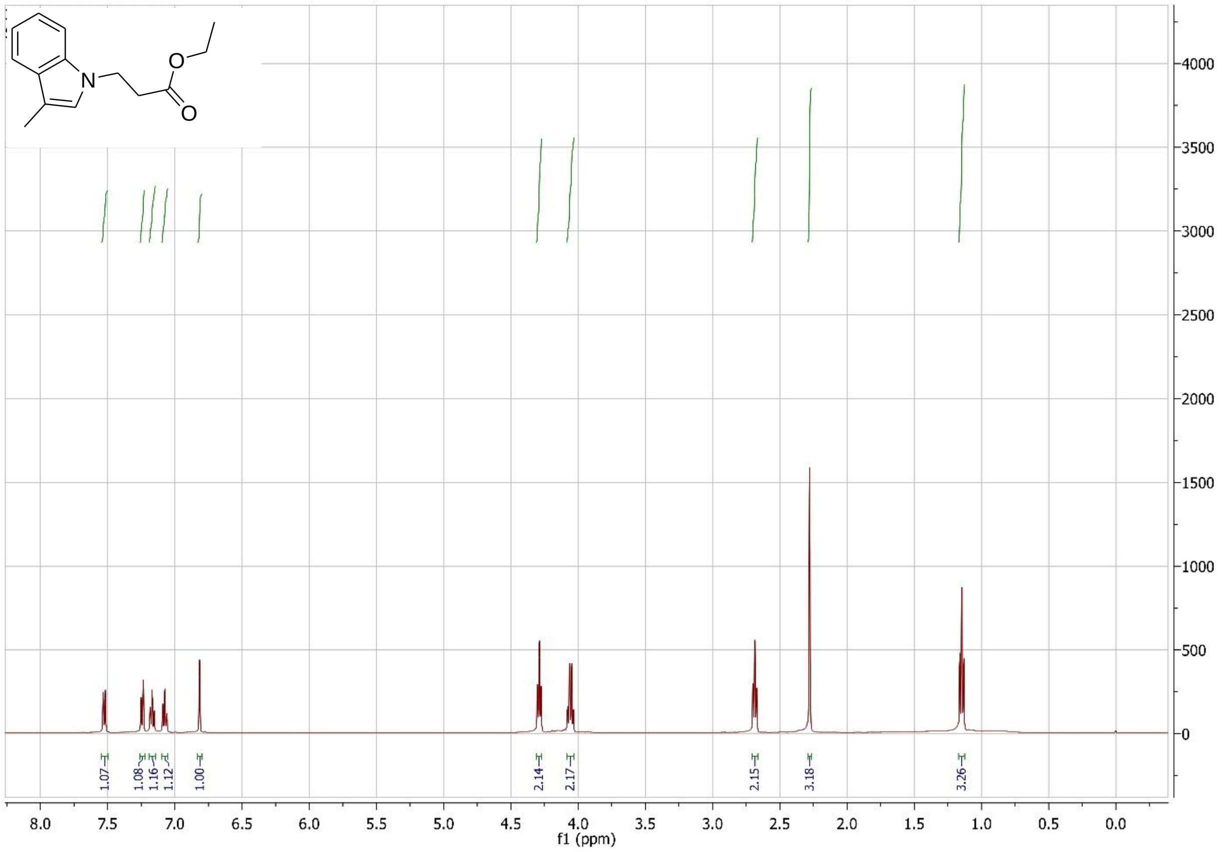

Figure S7. ^{1}H NMR spectrum of compound **16b.**

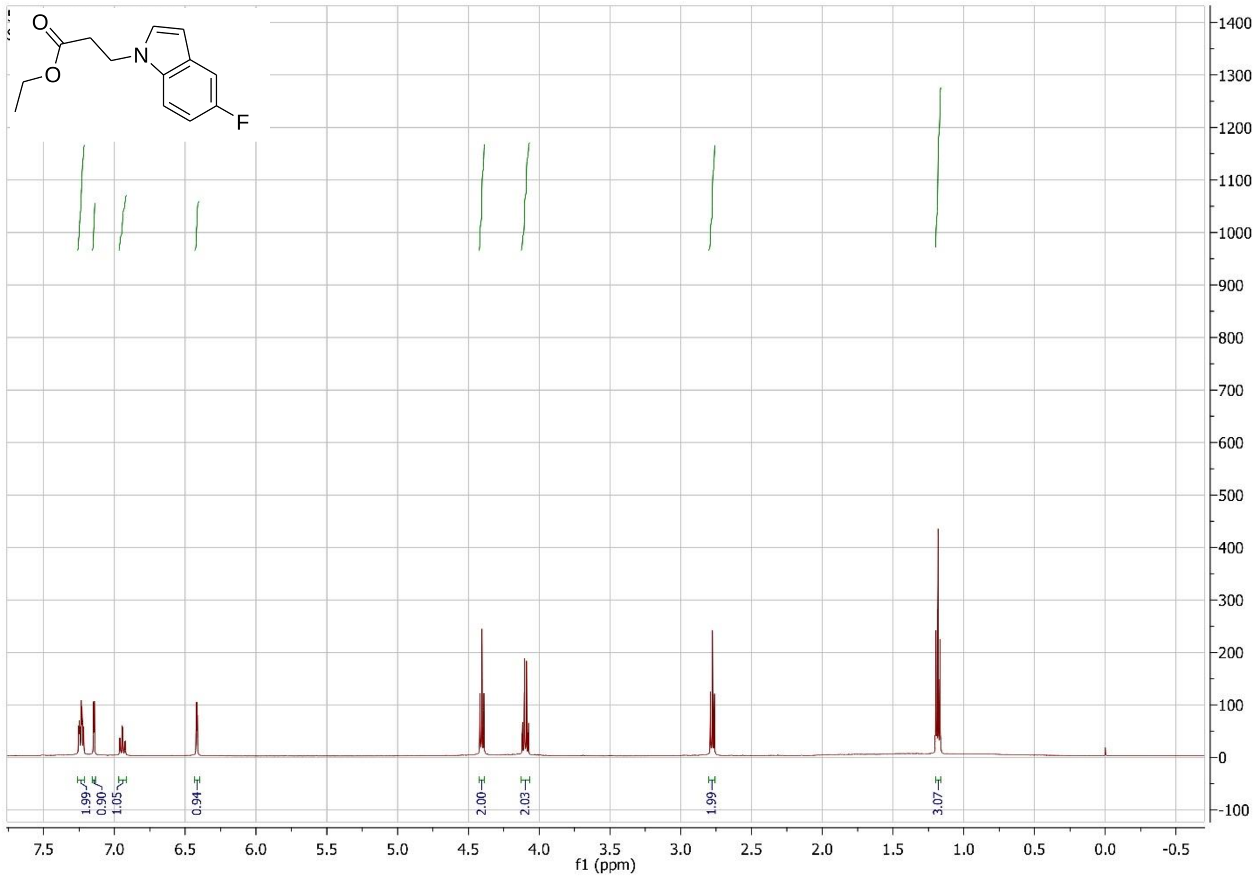

Figure S8. ^{1}H NMR spectrum of compound **17b.**

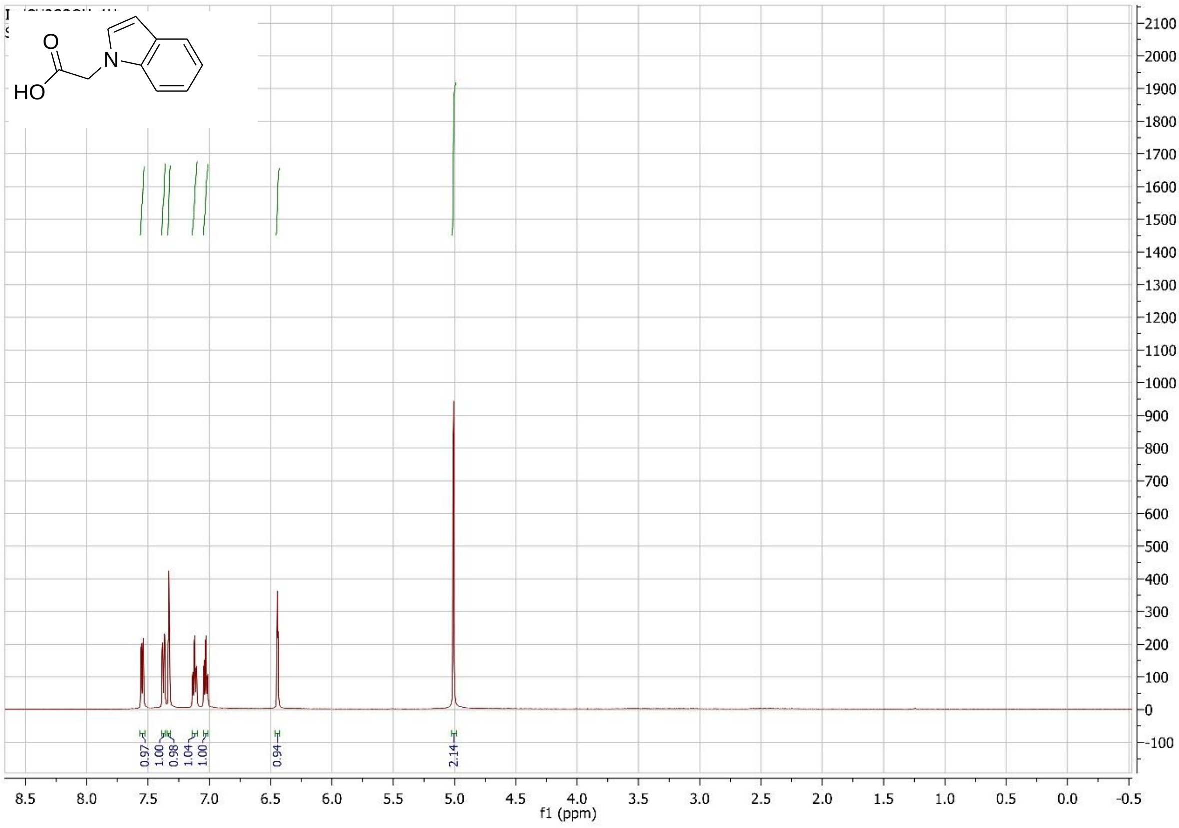

Figure S9. ^{1}H NMR spectrum of compound **18a.**

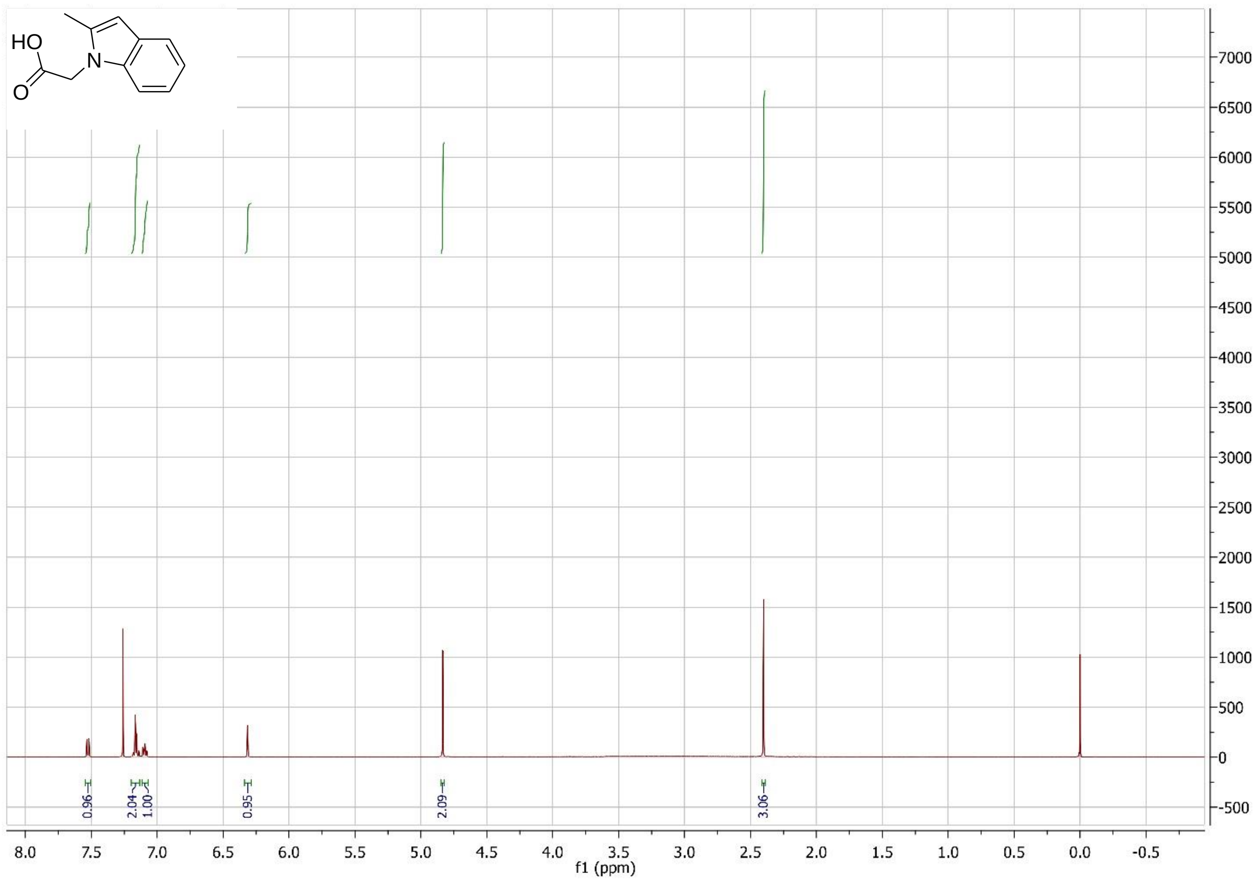

Figure S10. ^{1}H NMR spectrum of compound **19a**.

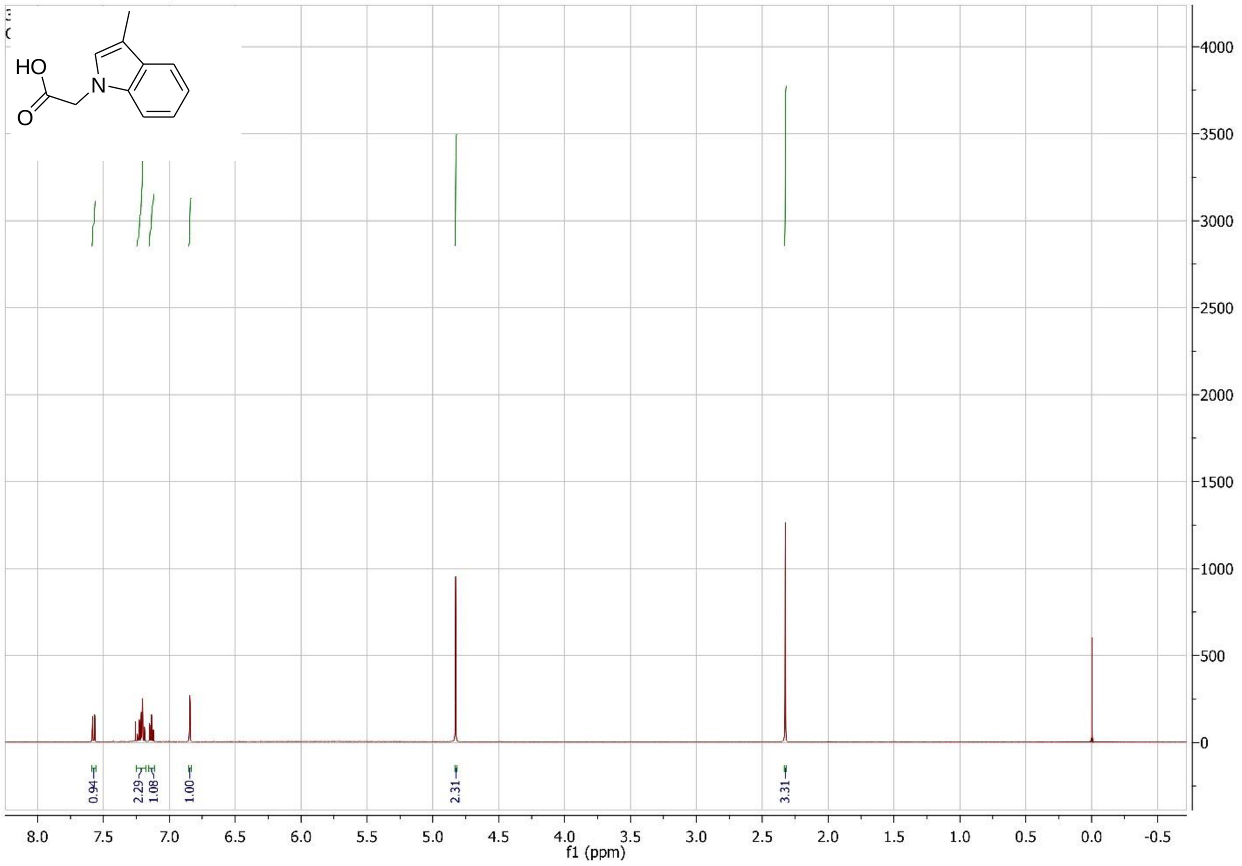

Figure S11. ^{1}H NMR spectrum of compound **20a.**

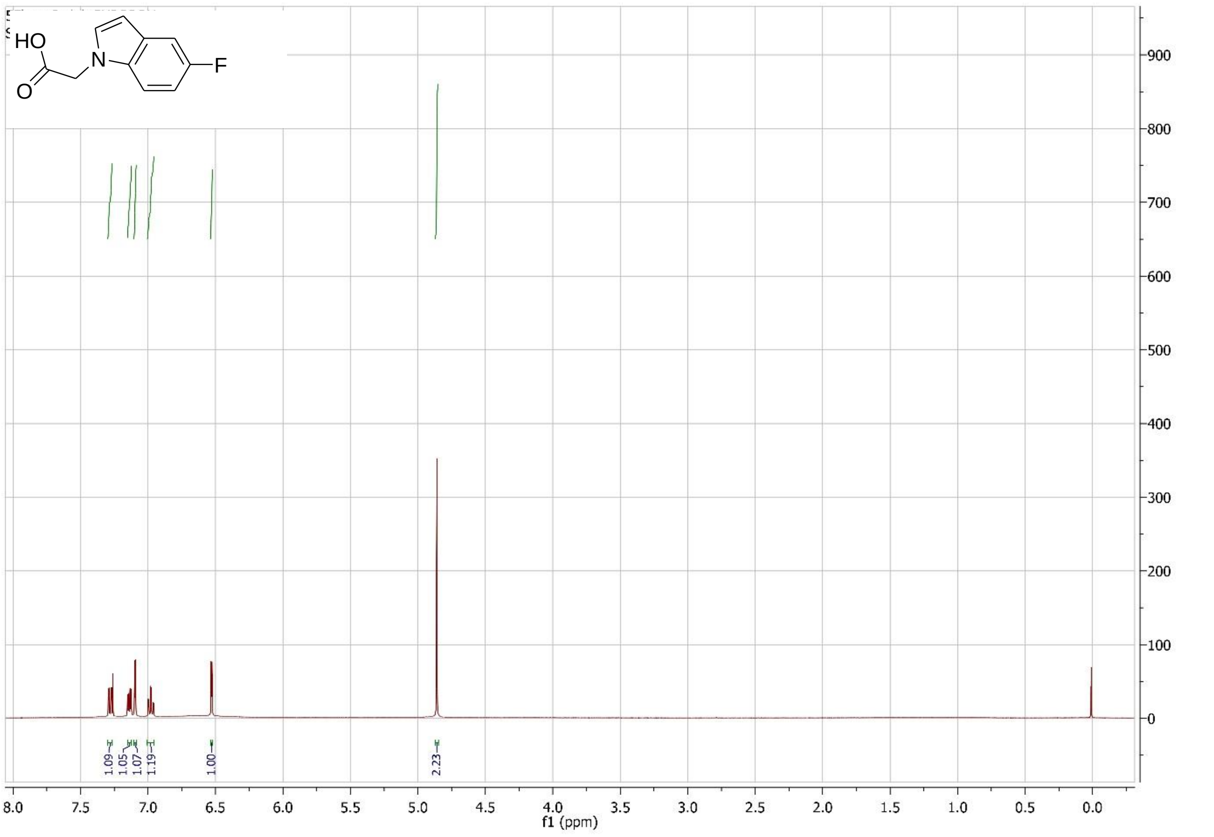

Figure S12. ^{1}H NMR spectrum of compound **21a**.

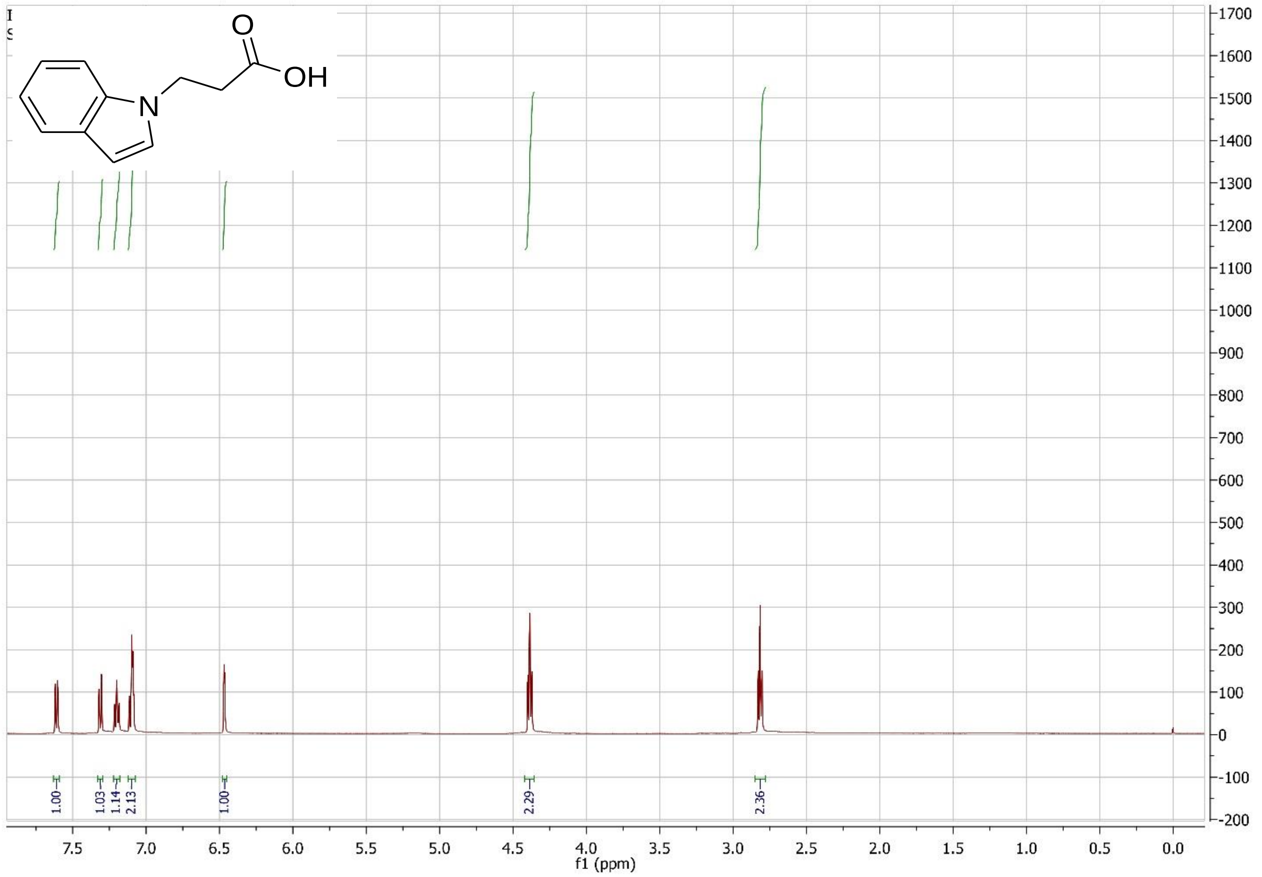

Figure S13. ^{1}H NMR spectrum of compound **18b.**

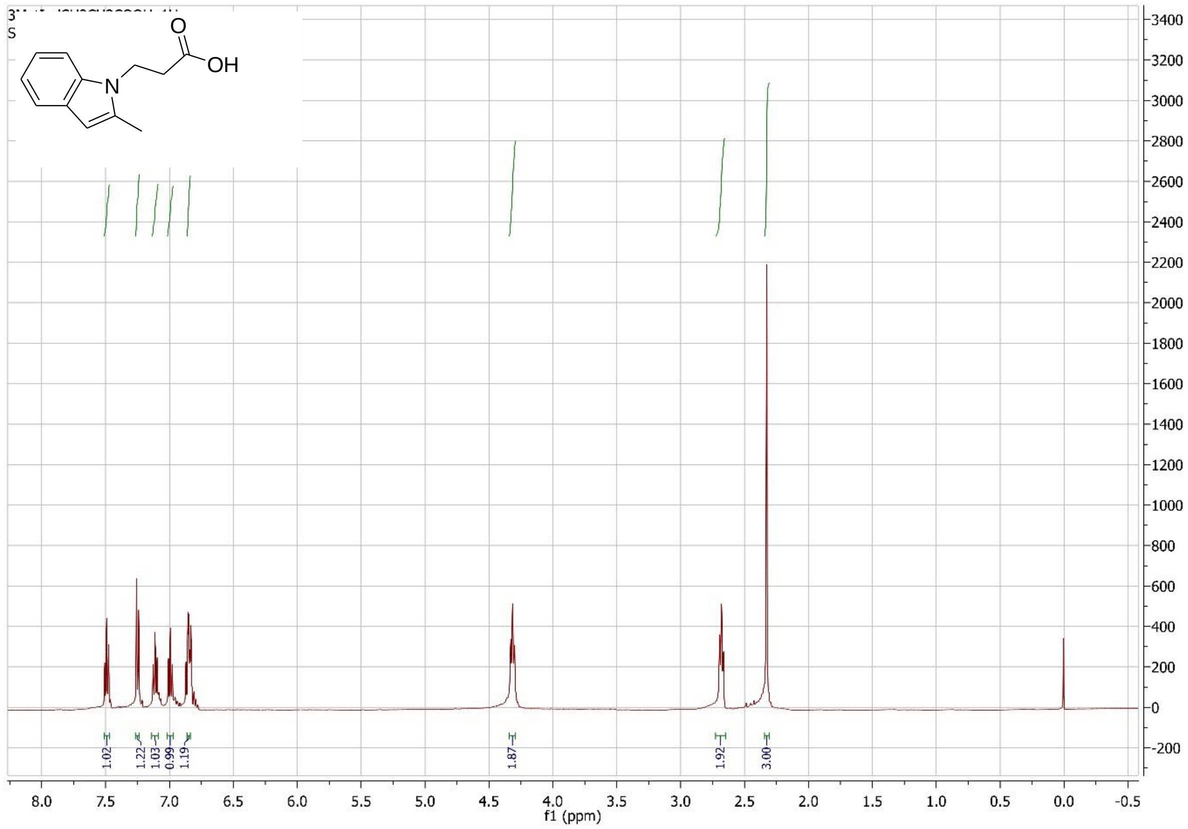

Figure S14. ^{1}H NMR spectrum of compound 1**9b.**

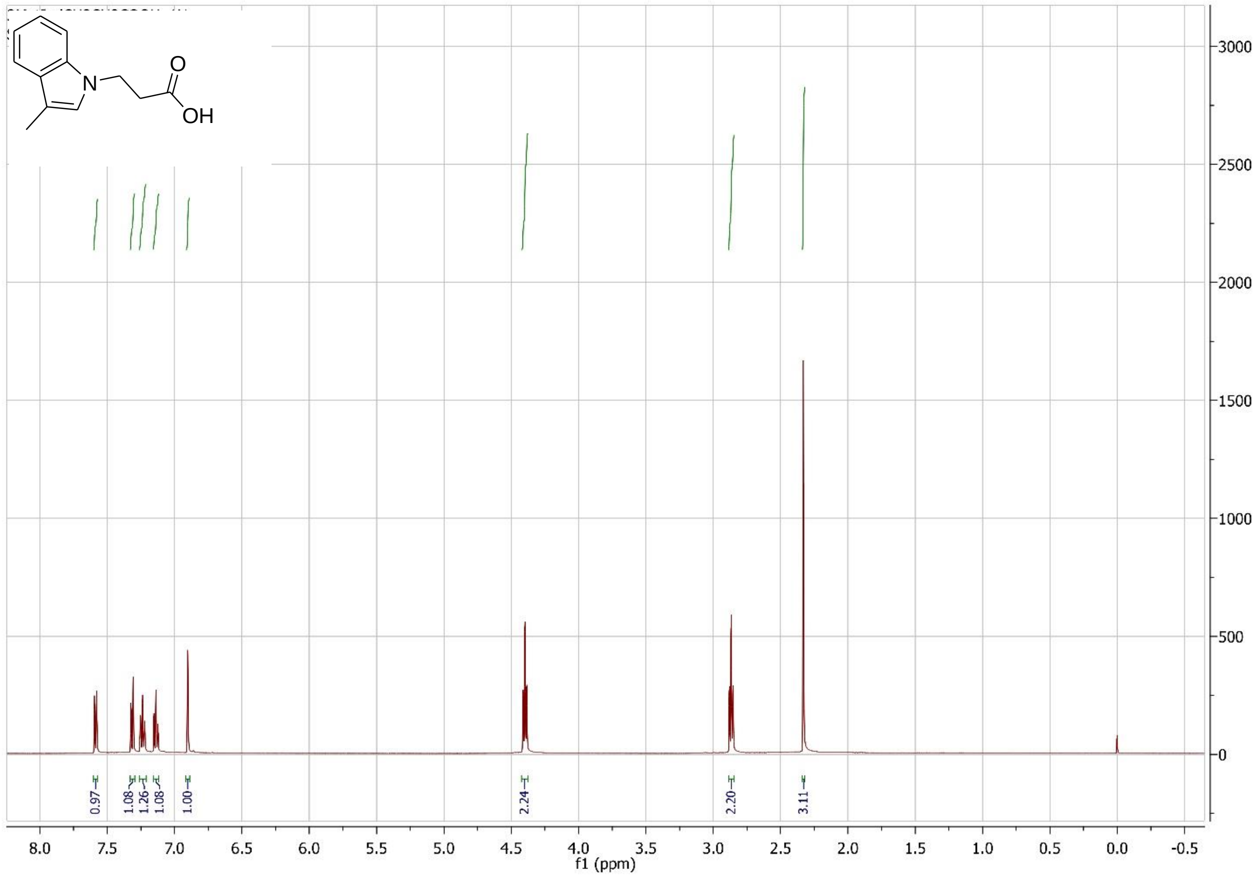

Figure S15. ^{1}H NMR spectrum of compound **20b.**

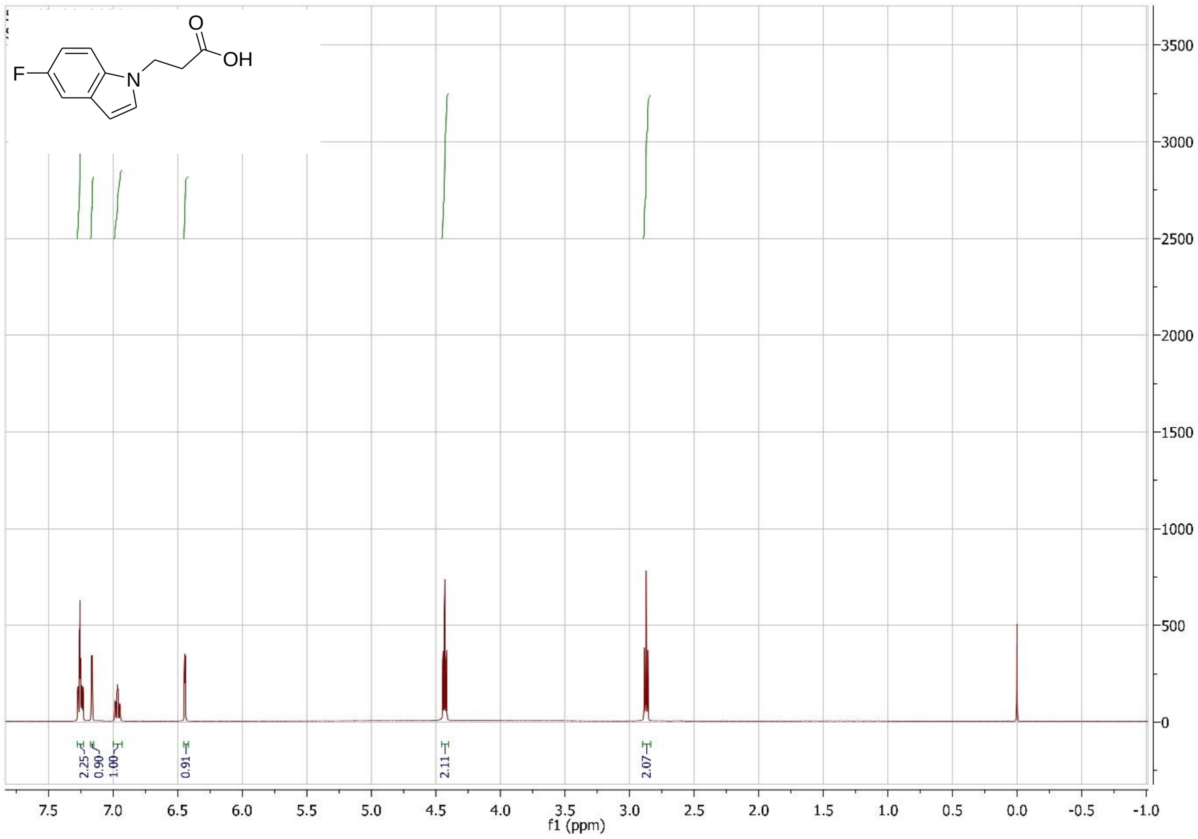

Figure S16. ^{1}H NMR spectrum of compound **21b.**

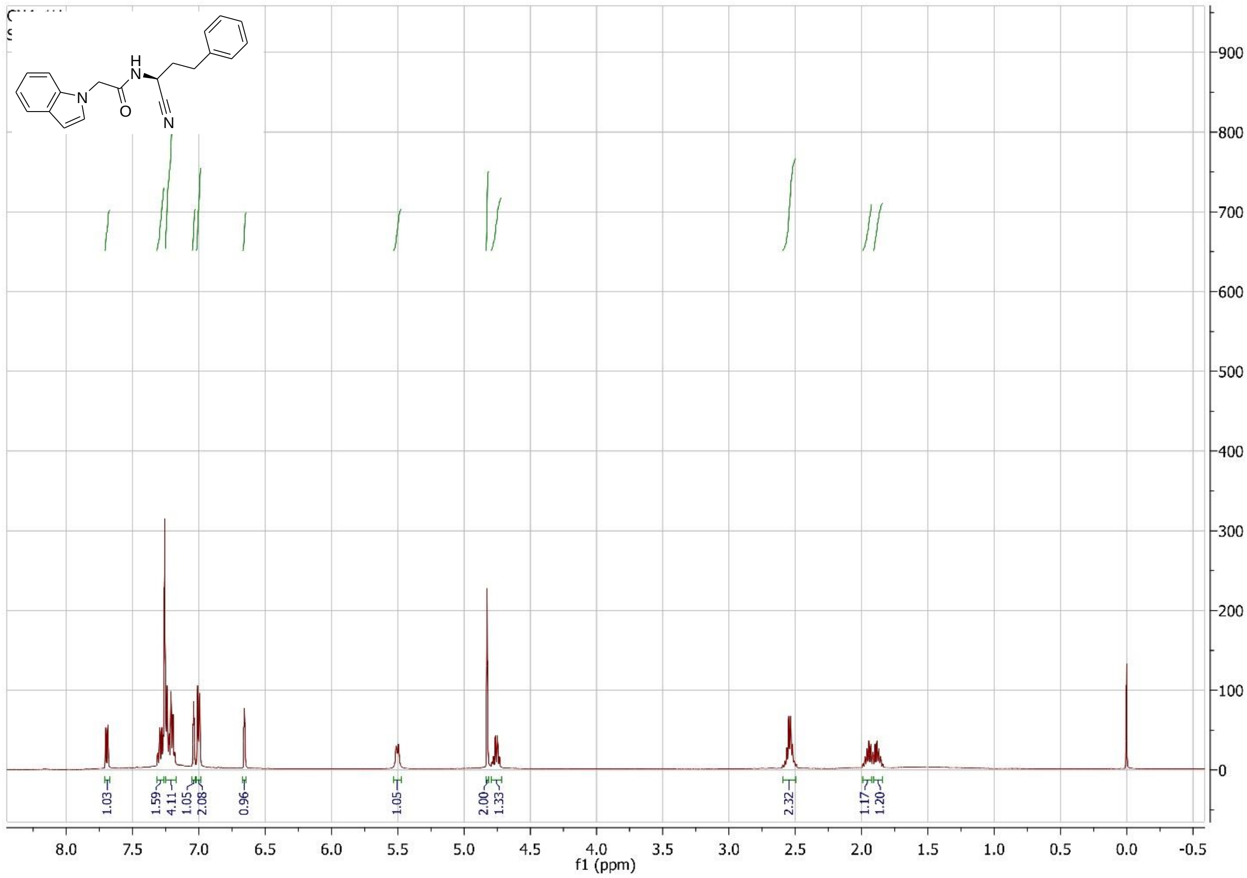

Figure S17. ^{1}H NMR spectrum of compound **1a.**

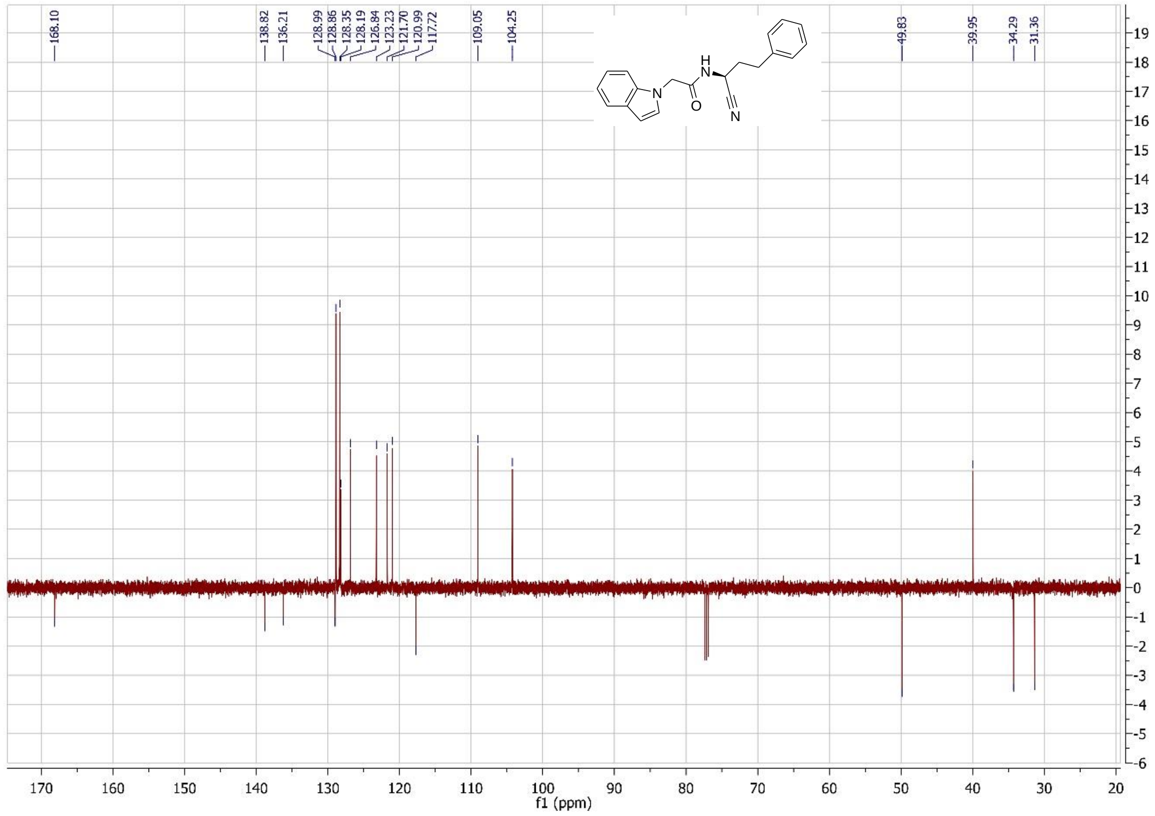

Figure S18. ^{13}C NMR spectrum of compound **1a.**

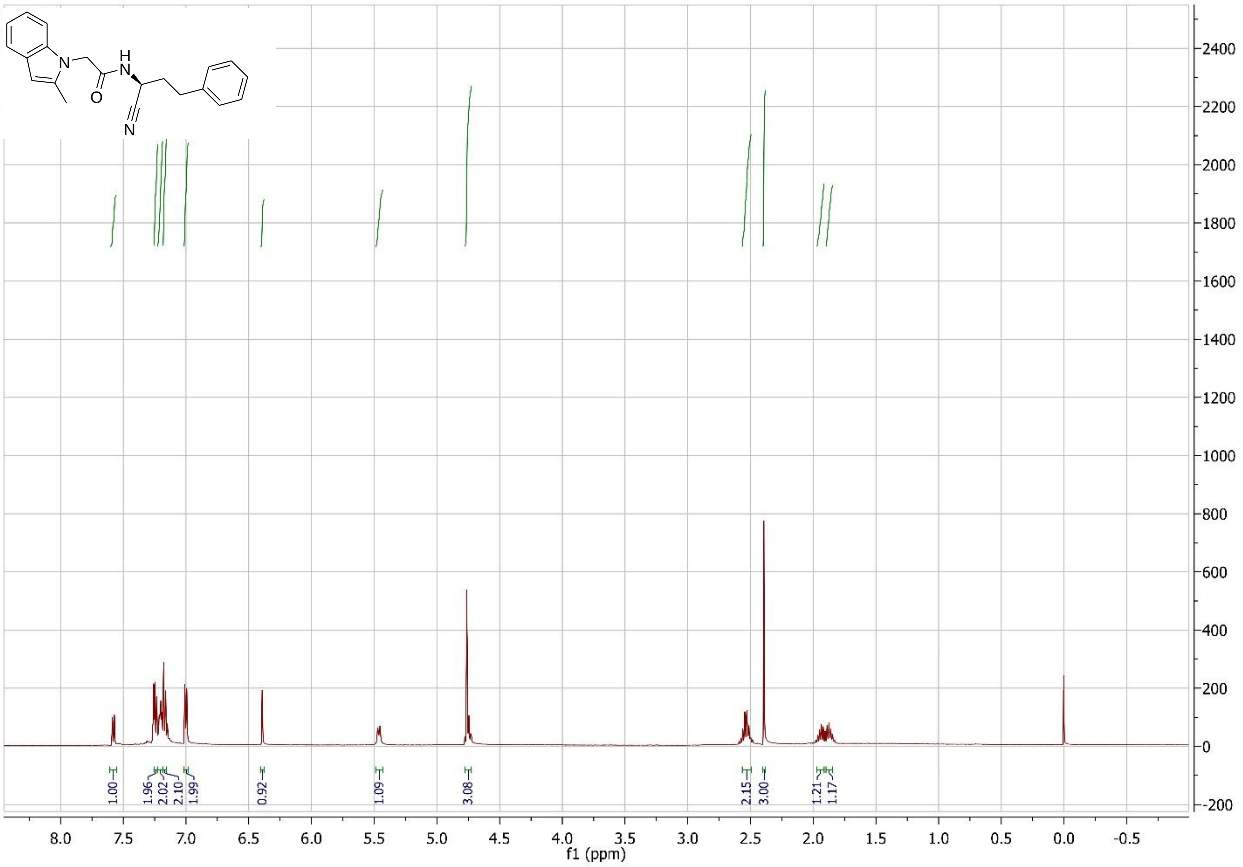

Figure S19. ^{1}H NMR spectrum of compound **2a.**

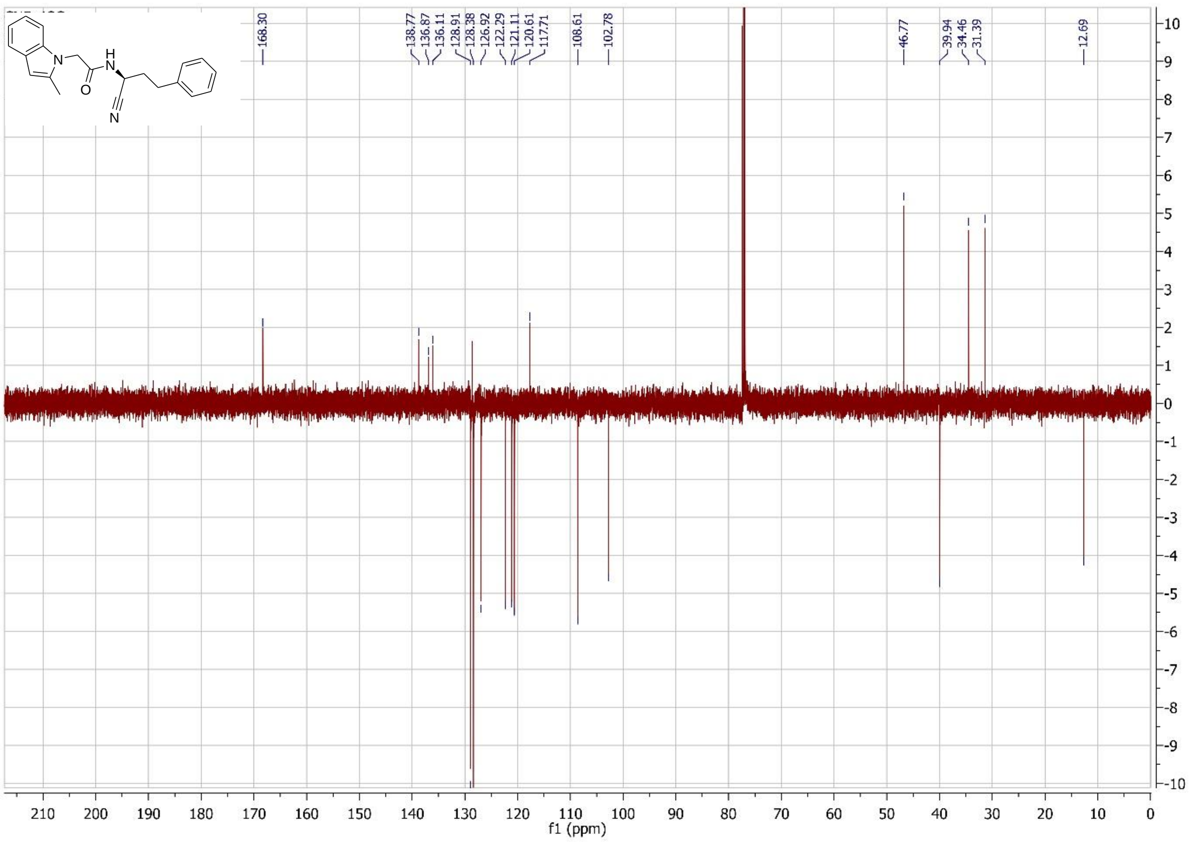

Figure S20. ^{13}C NMR spectrum of compound **2a**.

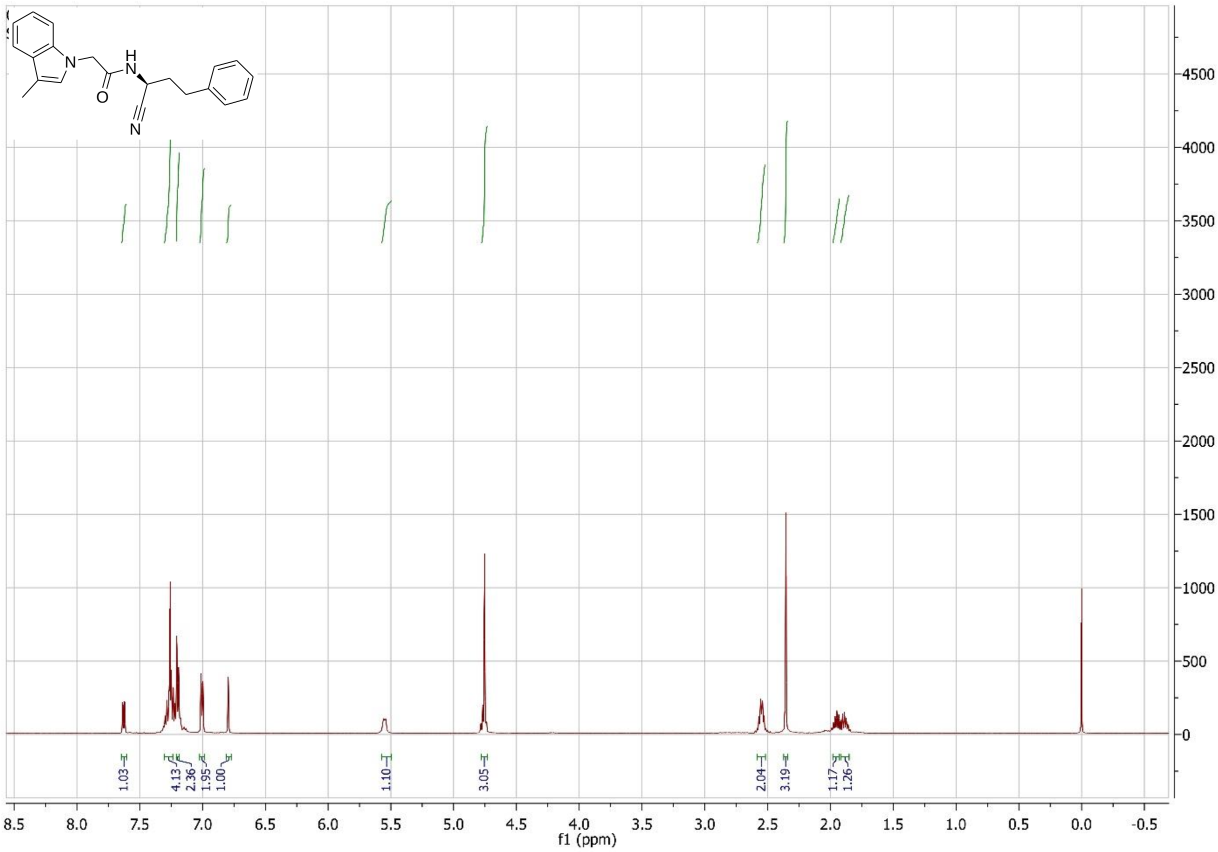

Figure S21. ^{1}H NMR spectrum of compound **3a.**

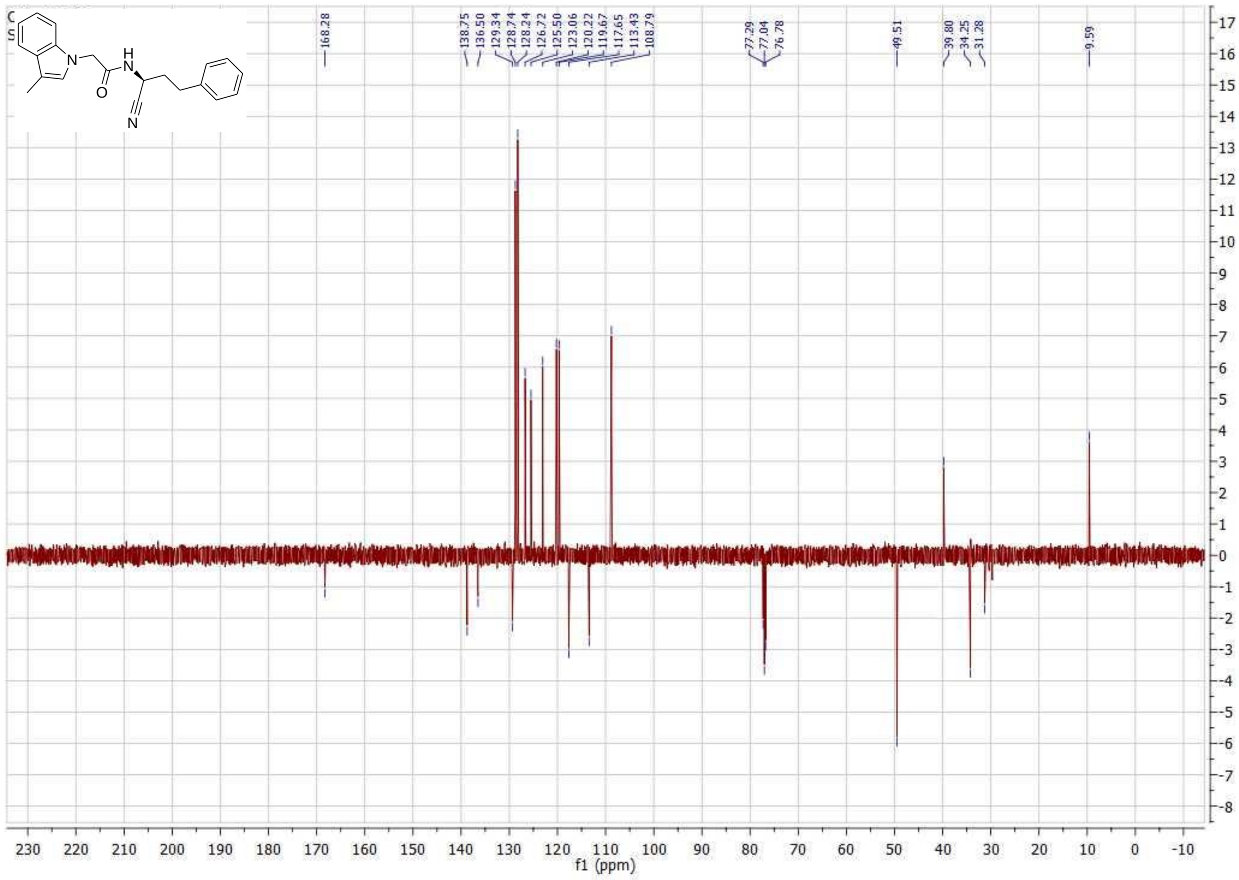

Figure S22. ^{13}C NMR spectrum of compound **3a**.

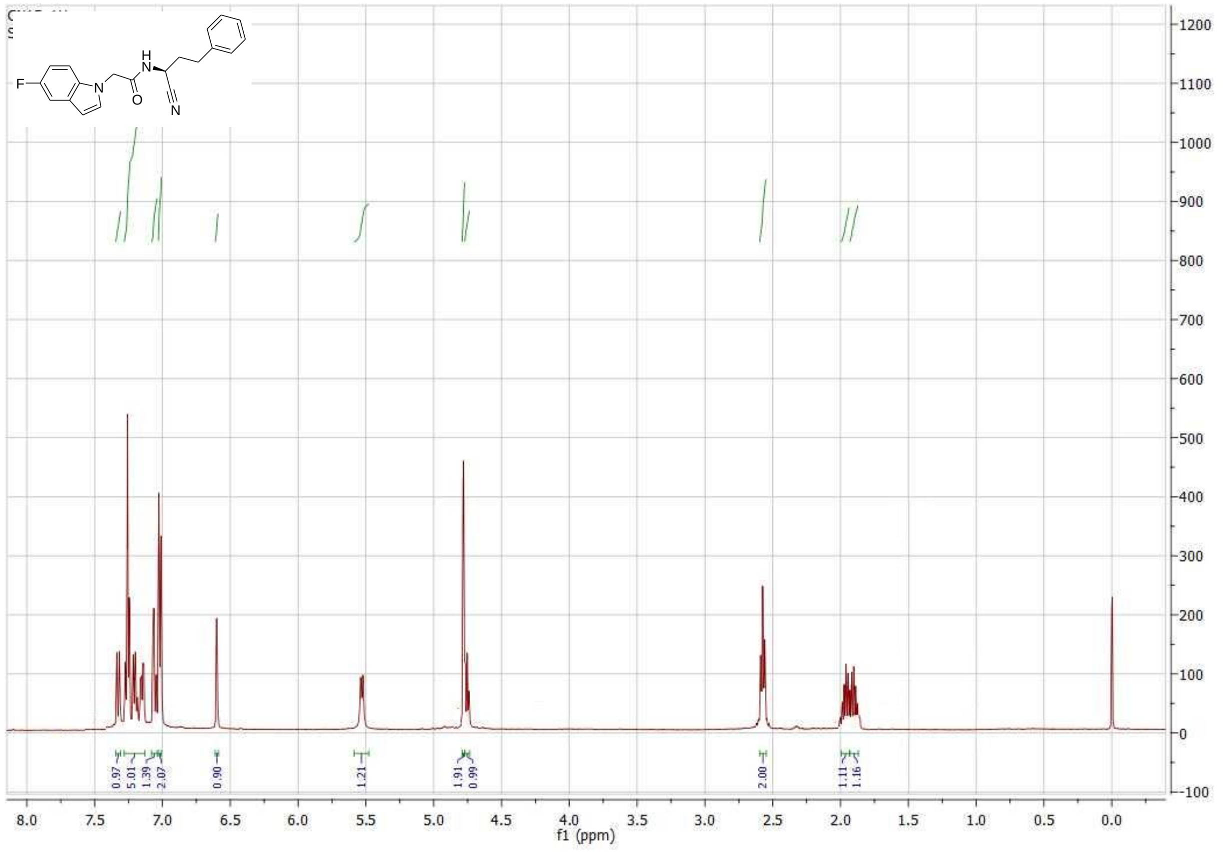

Figure S23. ^{1}H NMR spectrum of compound **4a.**

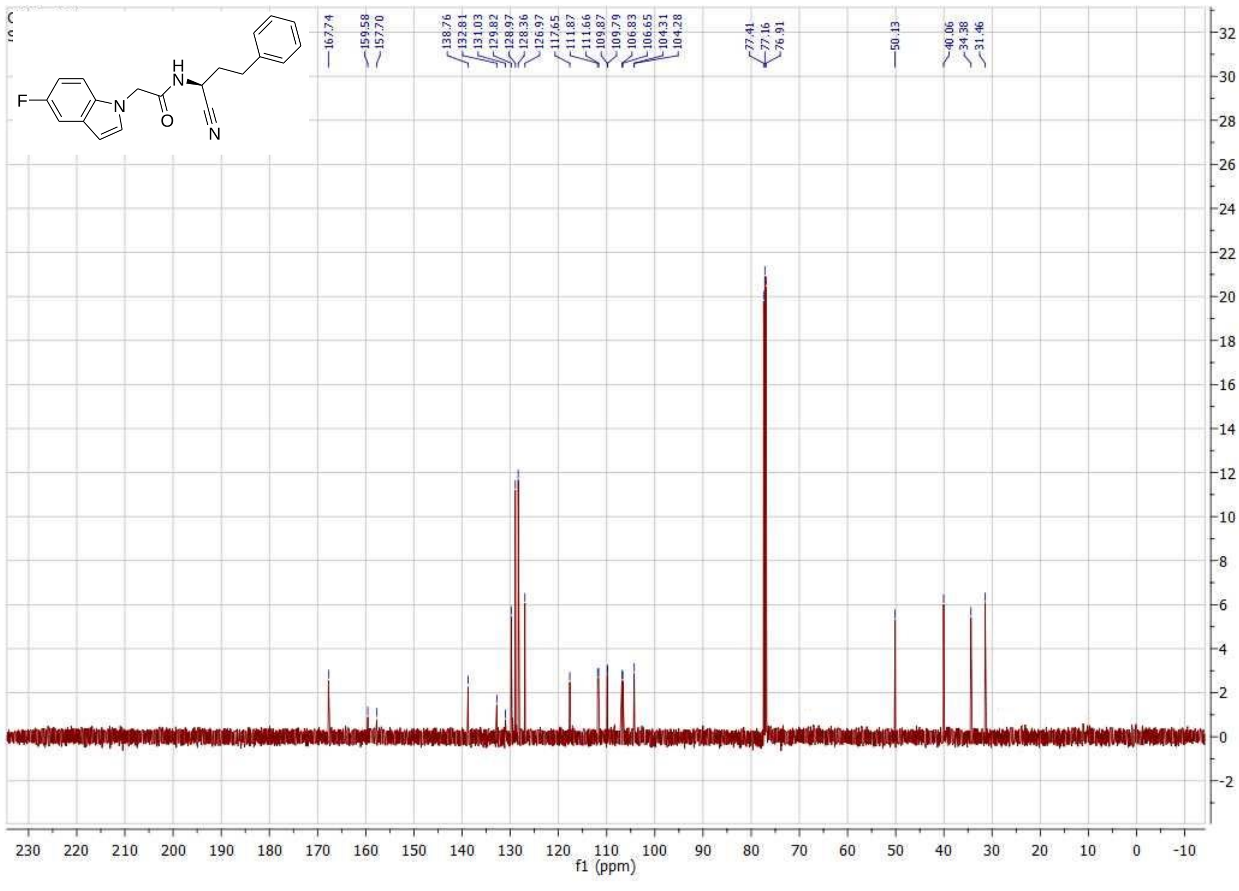

Figure S24. ^{13}C NMR spectrum of compound **4a**.

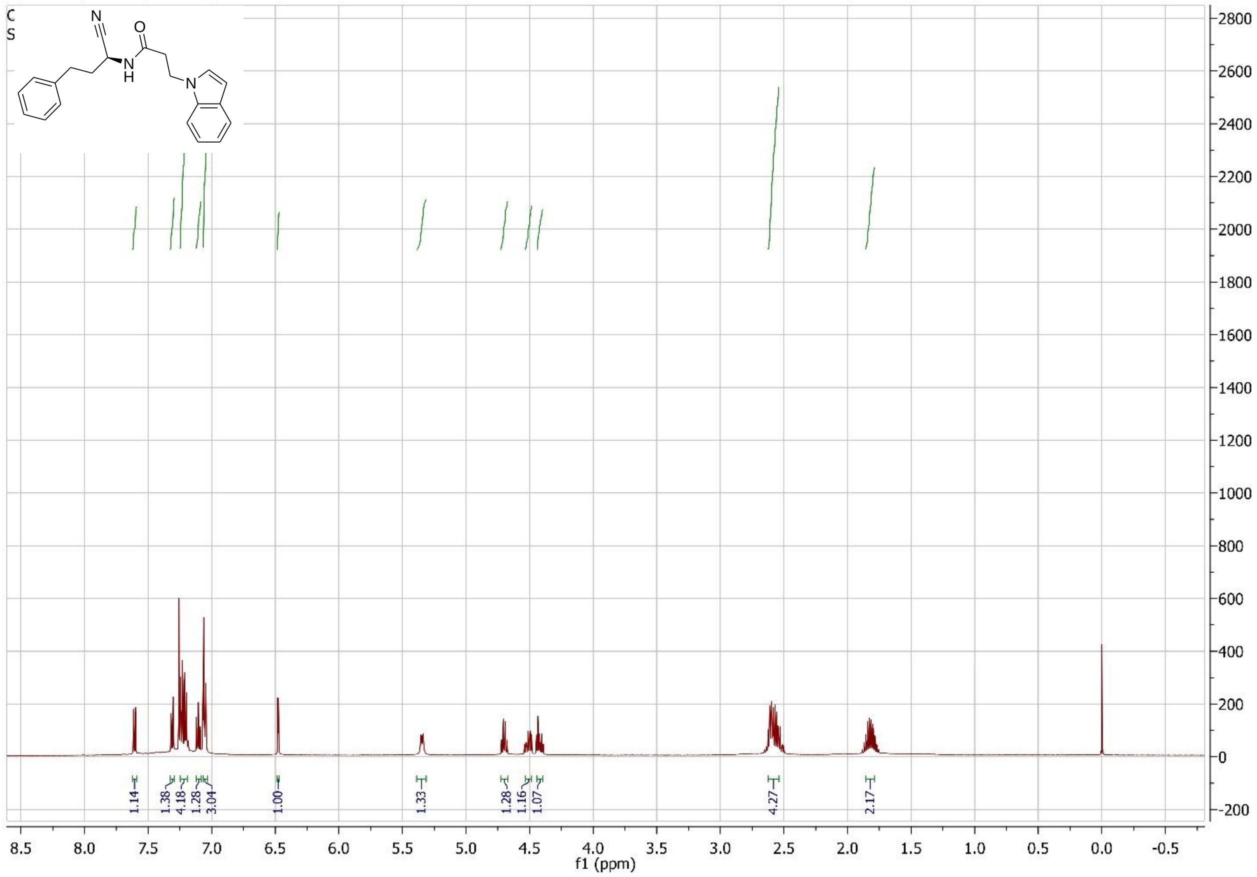

Figure S25. ^{1}H NMR spectrum of compound **1b.**

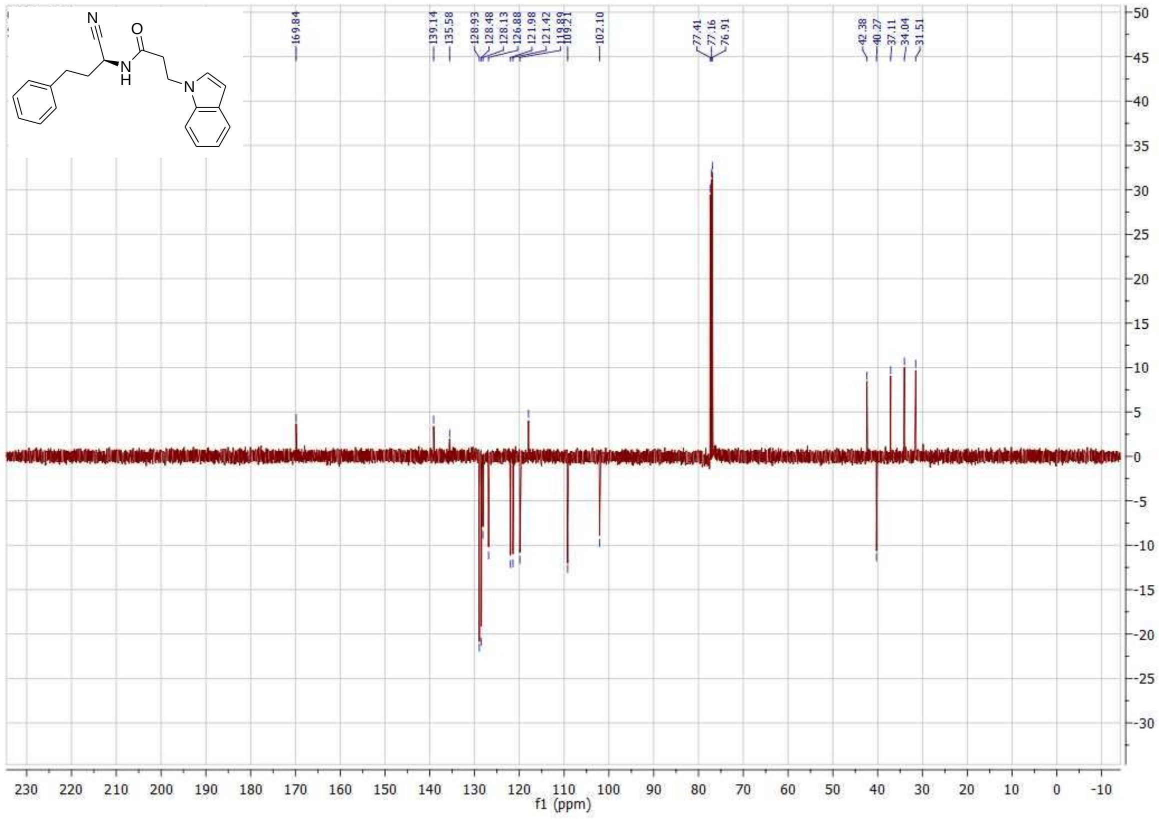

Figure S26. ^{13}C NMR spectrum of compound **1b**.

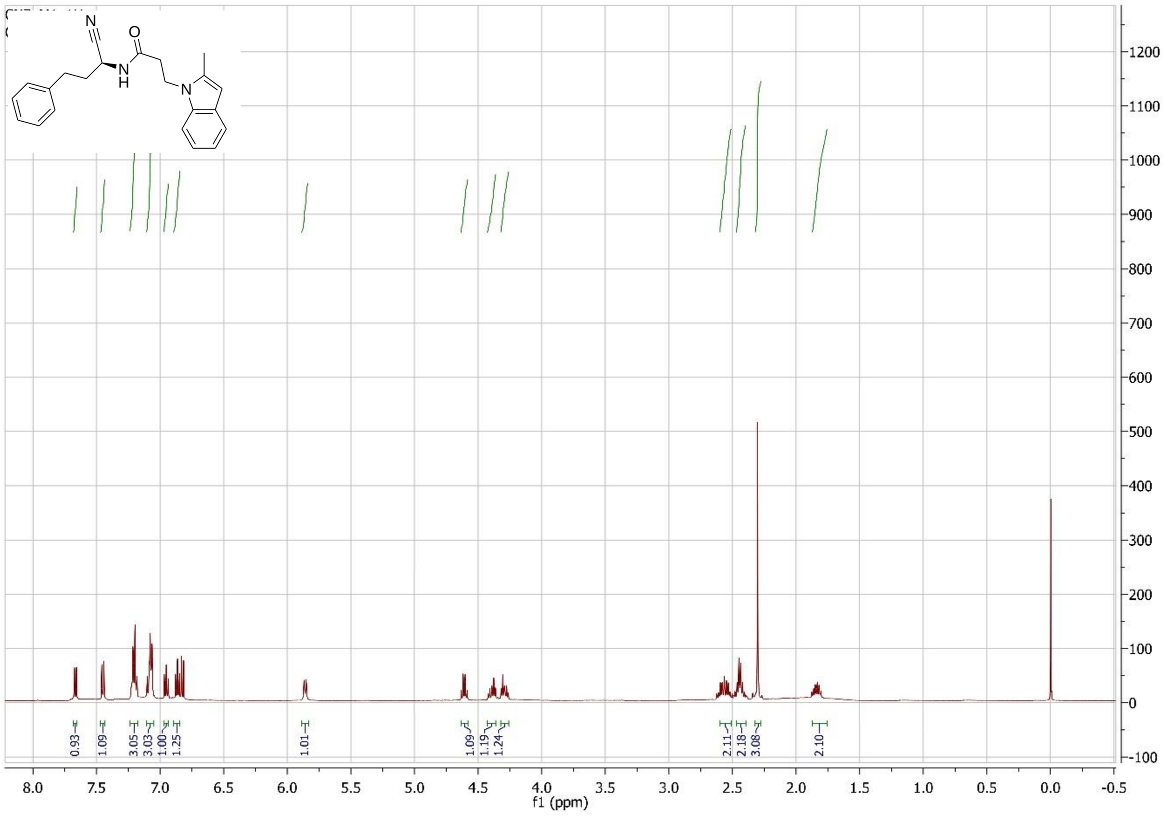

Figure S27. ^{1}H NMR spectrum of compound **2b.**

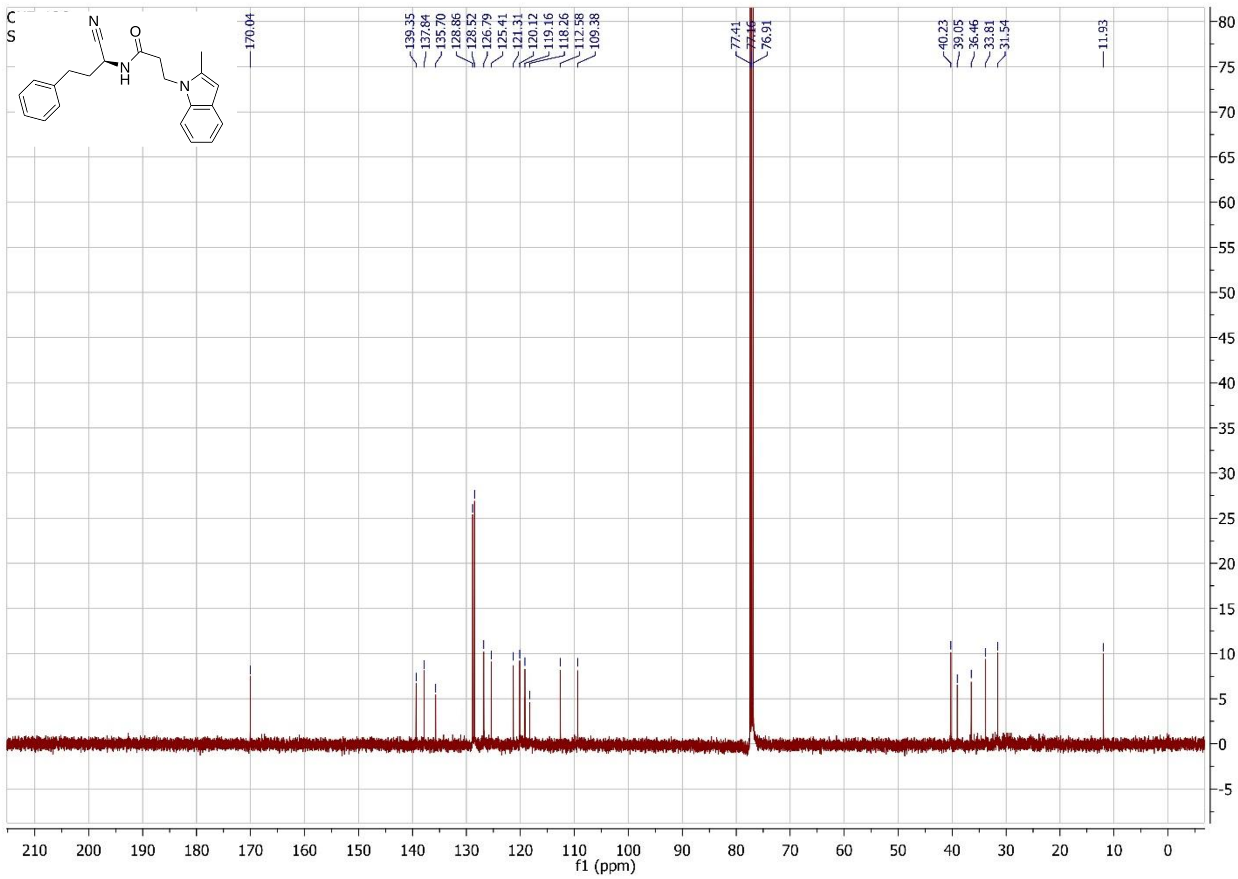

Figure S28. ^{13}C NMR spectrum of compound **2b**.

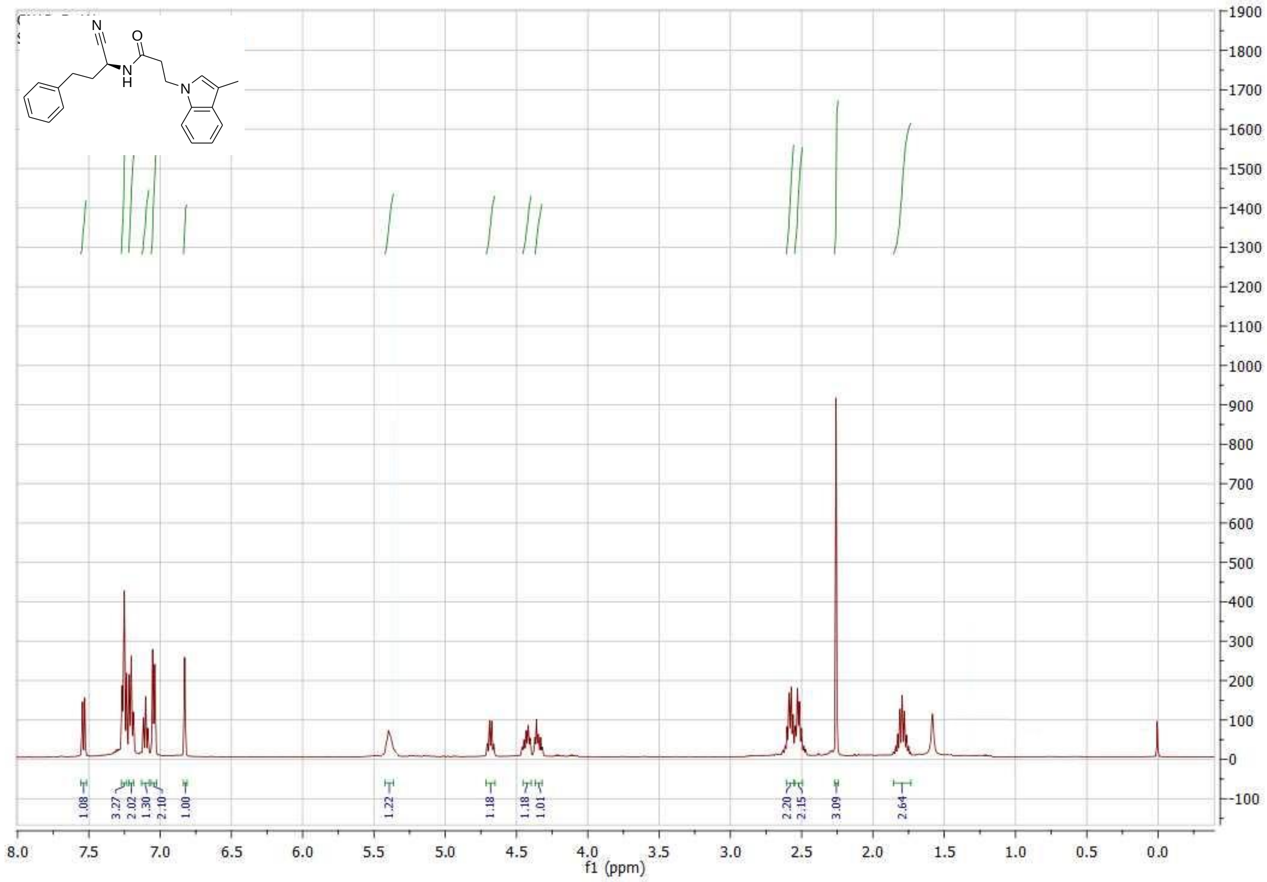

Figure S29. ^{1}H NMR spectrum of compound **3b.**

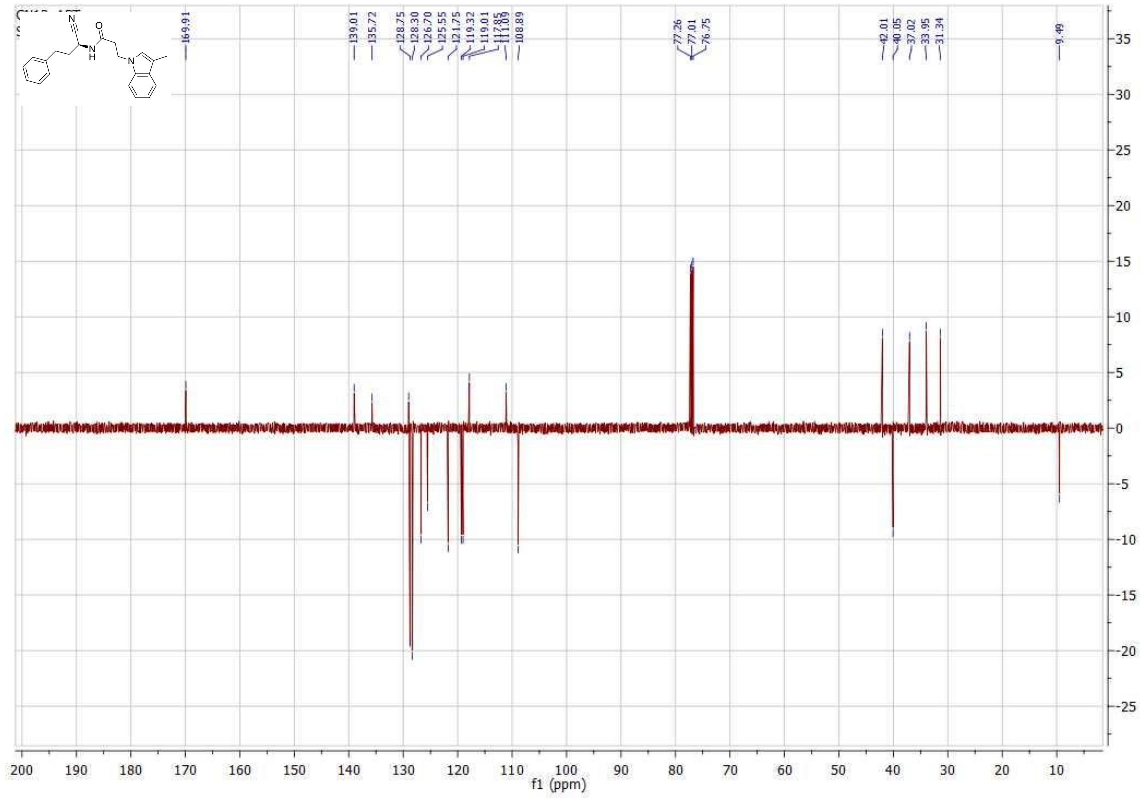

Figure S30. ^{13}C NMR spectrum of compound **3b**.

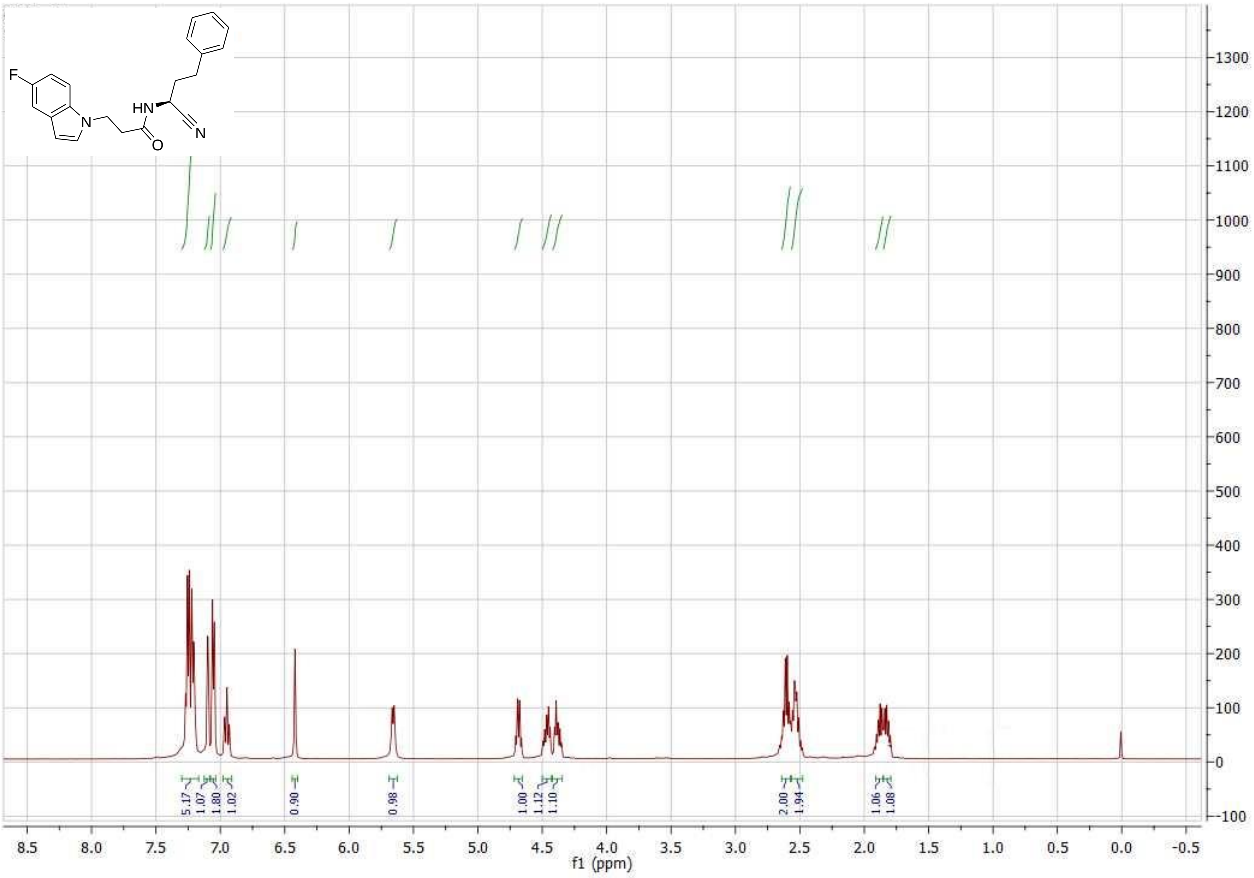

Figure S31. ^{1}H NMR spectrum of compound **4b.**

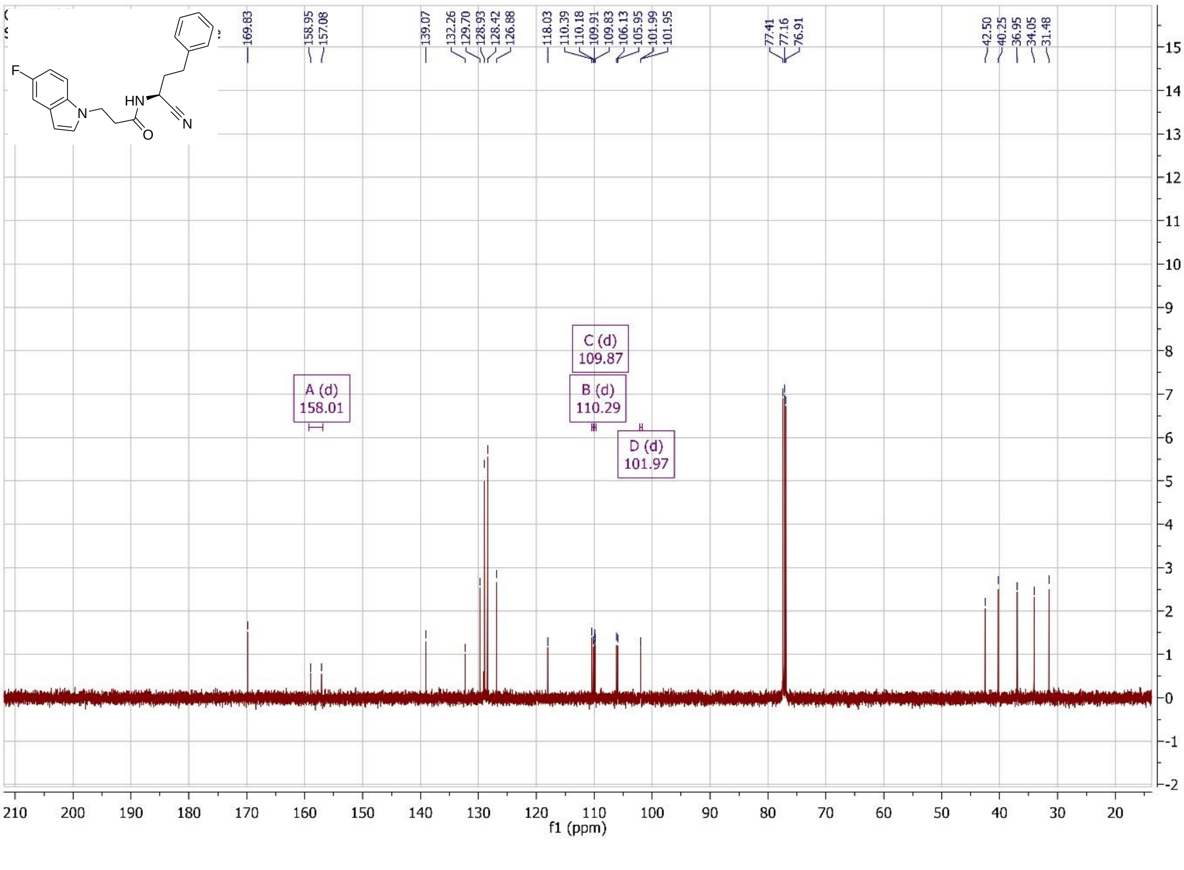

Figure S32. ^{13}C NMR spectrum of compound **4b**.

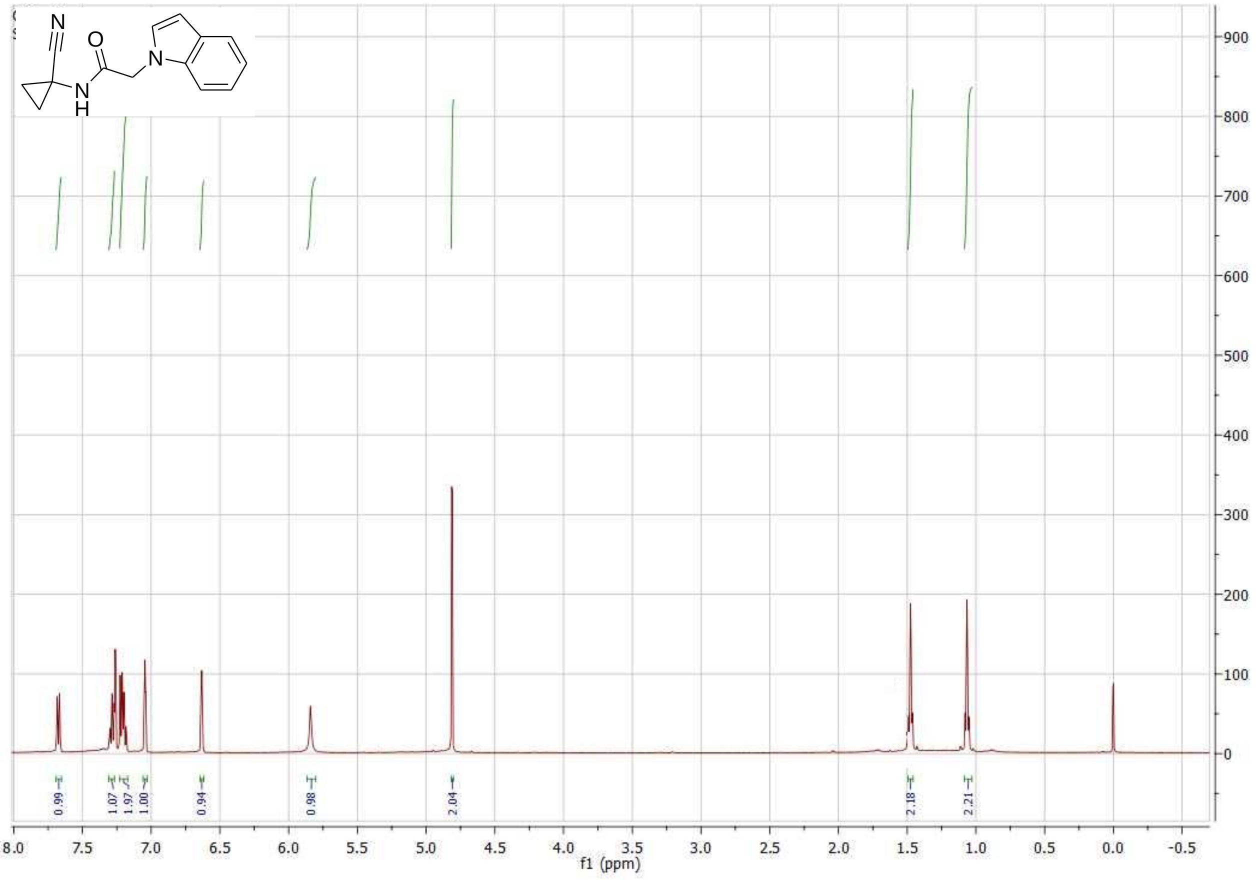

Figure S33. ^{1}H NMR spectrum of compound **5a.**

This report was created by ACD/NMR Processor Academic Edition. For more information go to www.acdlabs.com/nmrproc/

08/04/2024 14:51:28

Acquisition Time (sec)	1.0486	*Comment*	STANDARD 1H OBSERVE - profile			*Date*	Feb 20 2023
Date Stamp	Feb 20 2023	*File Name*	C:\Users\User\Desktop\Dottorato\Spettri\Spettri 2023\200223\CN1_APT.fid\fid				
Frequency (MHz)	125.67	*Nucleus*	13C	*Number of Transients*	920	*Original Points Count*	32768
Points Count	32768	*Pulse Sequence*	APT0	*Receiver Gain*	30.00	*Solvent*	DEUTERIUM OXIDE
Spectrum Offset (Hz)	14142.4297	*Spectrum Type*	APT	*Sweep Width (Hz)*	31250.00	*Temperature (degree C)*	25.000

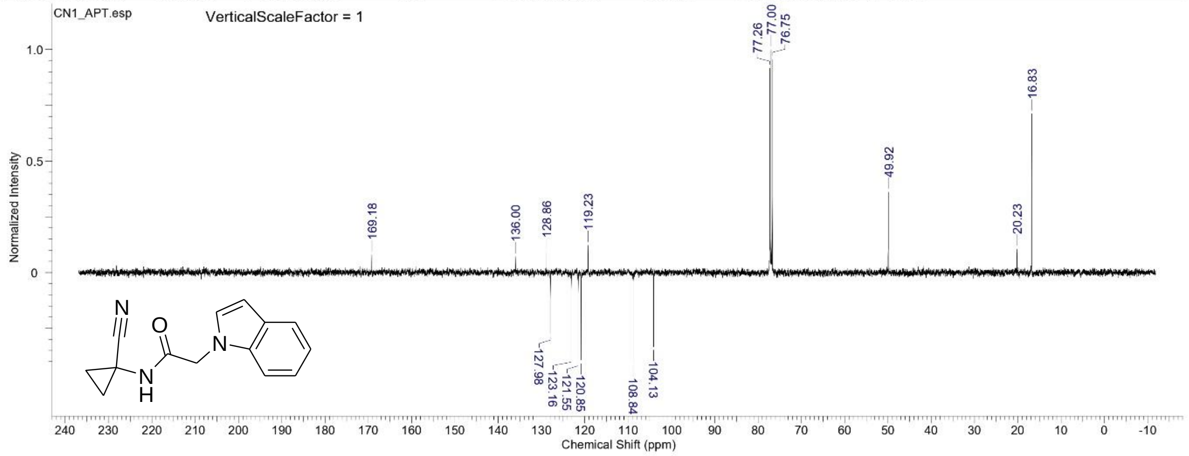

No.	(ppm)	(Hz)	Height	No.	(ppm)	(Hz)	Height	No.	(ppm)	(Hz)	Height	No.	(ppm)	(Hz)	Height
1	16.83	2114.8	0.7116	5	77.00	9676.7	1.0000	9	119.23	14984.1	0.1233	13	127.98	16083.7	-0.2734
2	20.23	2542.1	0.1066	6	77.26	9709.1	0.9183	10	120.85	15187.2	-0.3951	14	128.86	16194.3	0.0896
3	49.92	6273.9	0.3618	7	104.13	13086.2	-0.3339	11	121.55	15275.9	-0.3890	15	136.00	17091.8	0.0712
4	76.75	9645.2	0.9572	8	108.84	13678.5	-0.4070	12	123.16	15478.1	-0.3733	16	169.18	21261.4	0.0795

Figure S34. ^{13}C NMR spectrum of compound **5a**.

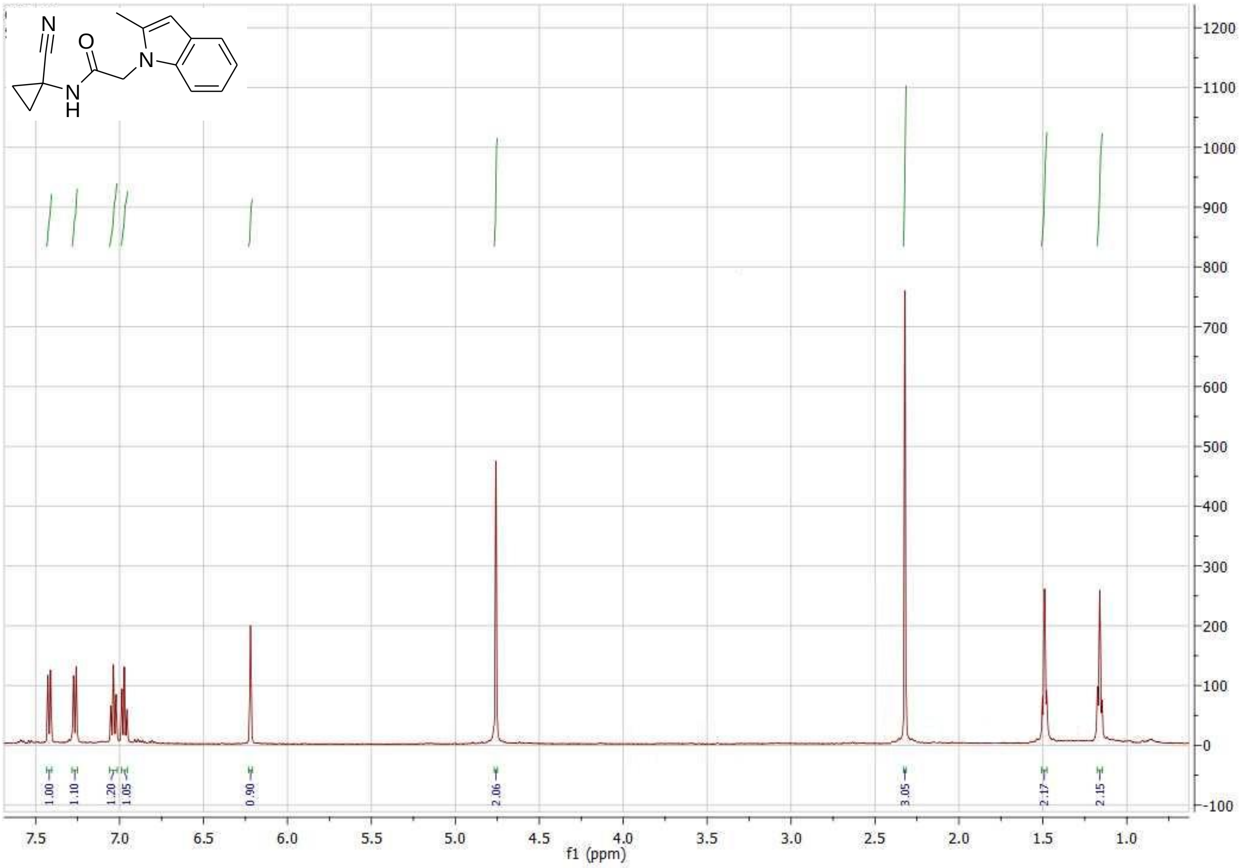

Figure S35. ^{1}H NMR spectrum of compound **6a.**

This report was created by ACD/NMR Processor Academic Edition. For more information go to www.acdlabs.com/nmrproc/

08/04/2024 14:33:42

Acquisition Time (sec)	1.0486	*Comment*	STANDARD 1H OBSERVE - profile			*Date*	Mar 2 2023
Date Stamp	Mar 2 2023	*File Name*	C:\Users\User\Desktop\Dottorato\Spettri\Spettri 2023\020323\CN2_APT.fid\fid				
Frequency (MHz)	125.67	*Nucleus*	13C	*Number of Transients*	1188	*Original Points Count*	32768
Points Count	32768	*Pulse Sequence*	APT0	*Receiver Gain*	30.00	*Solvent*	DEUTERIUM OXIDE
Spectrum Offset (Hz)	13509.9902	*Spectrum Type*	APT	*Sweep Width (Hz)*	31250.00	*Temperature (degree C)*	25.000

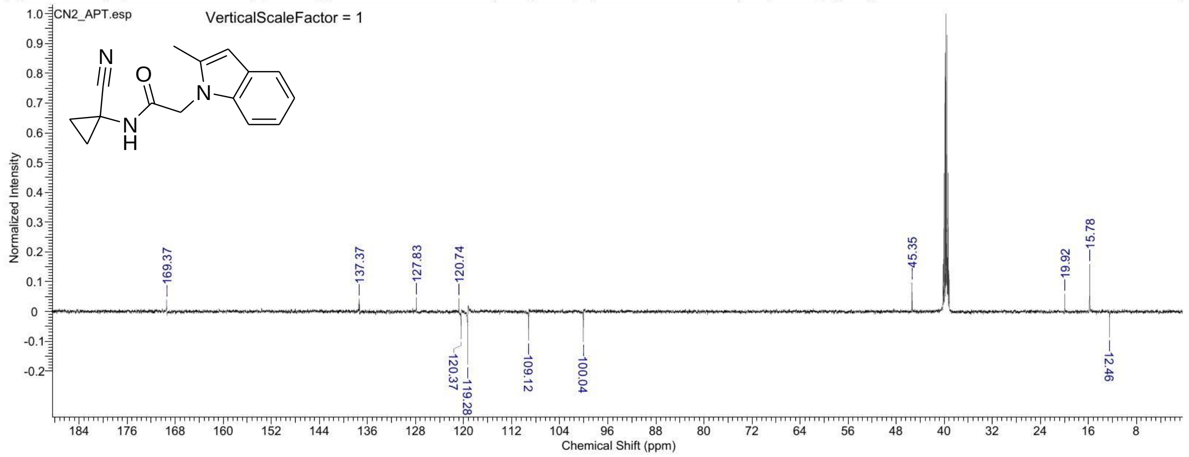

No.	(ppm)	(Hz)	Height	No.	(ppm)	(Hz)	Height	No.	(ppm)	(Hz)	Height	No.	(ppm)	(Hz)	Height	No.	(ppm)	(Hz)	Height
1	12.46	1565.3	-0.0873	4	45.35	5699.6	0.0970	7	119.28	14989.7	-0.1780	10	127.83	16064.5	0.0466	13	169.37	21285.1	0.0393
2	15.78	1983.1	0.1582	5	100.04	12572.0	-0.1030	8	120.37	15127.0	-0.0933	11	137.32	17257.6	0.0259				
3	19.92	2503.8	0.0588	6	109.12	13713.6	-0.0975	9	120.74	15173.7	0.0437	12	137.37	17263.3	0.0430				

Figure S36. ^{13}C NMR spectrum of compound **6a**.

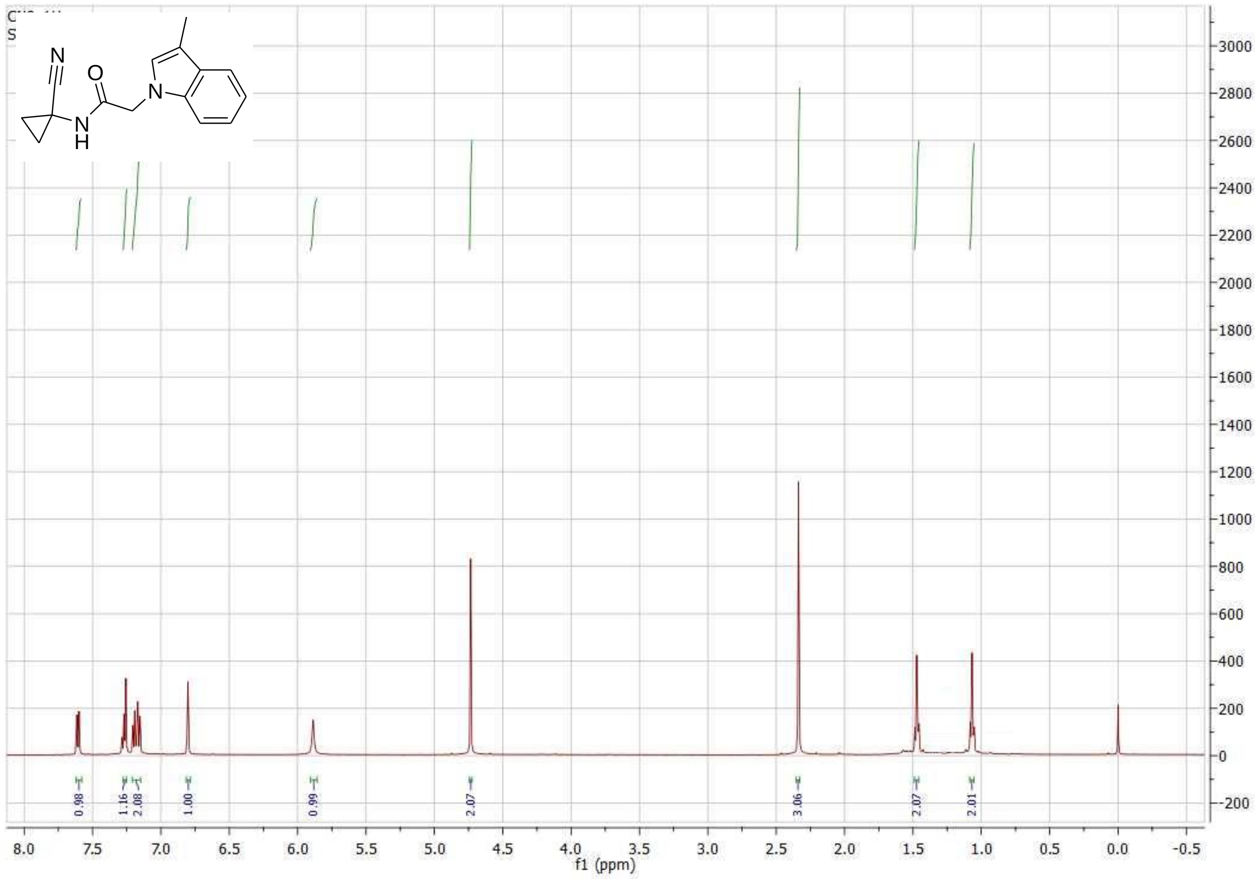

Figure S37. ^{1}H NMR spectrum of compound **7a.**

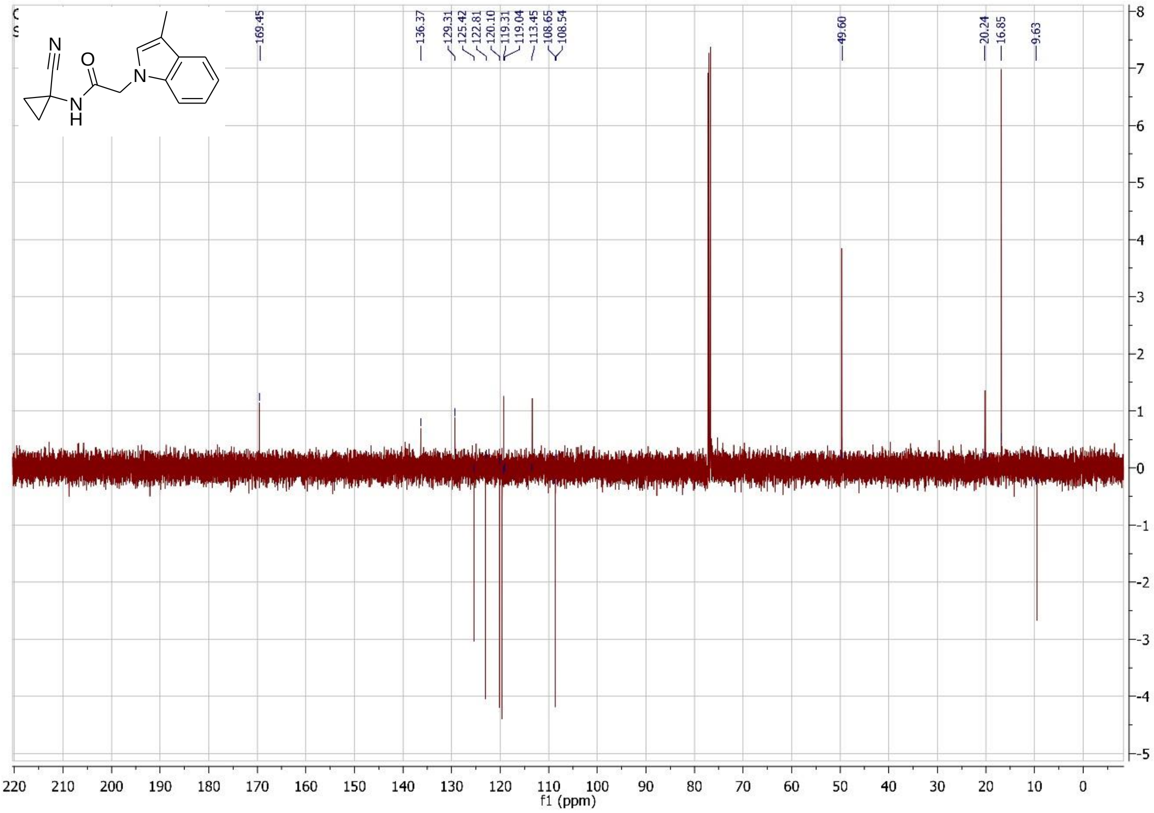

Figure S38. ^{13}C NMR spectrum of compound **7a**.

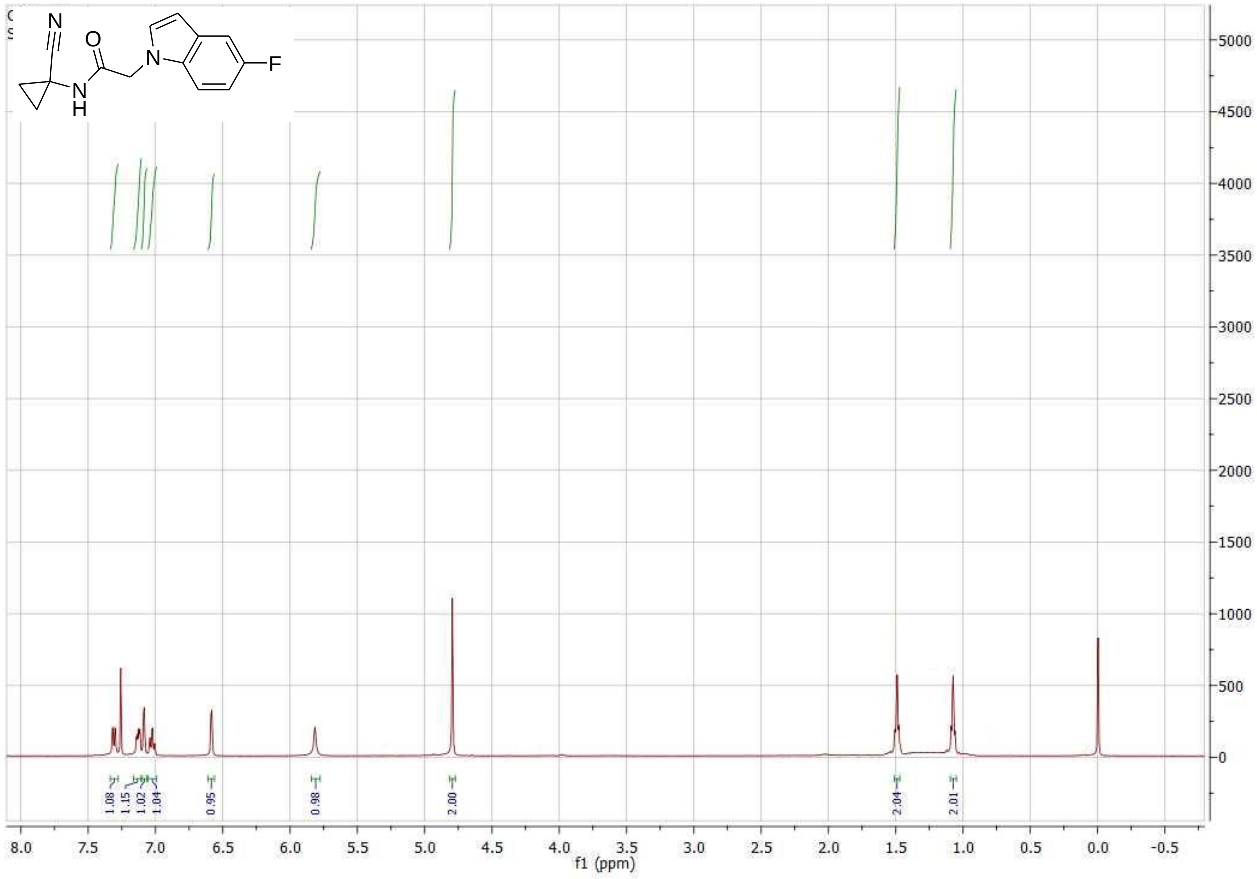

Figure S39. ^{1}H NMR spectrum of compound **8a.**

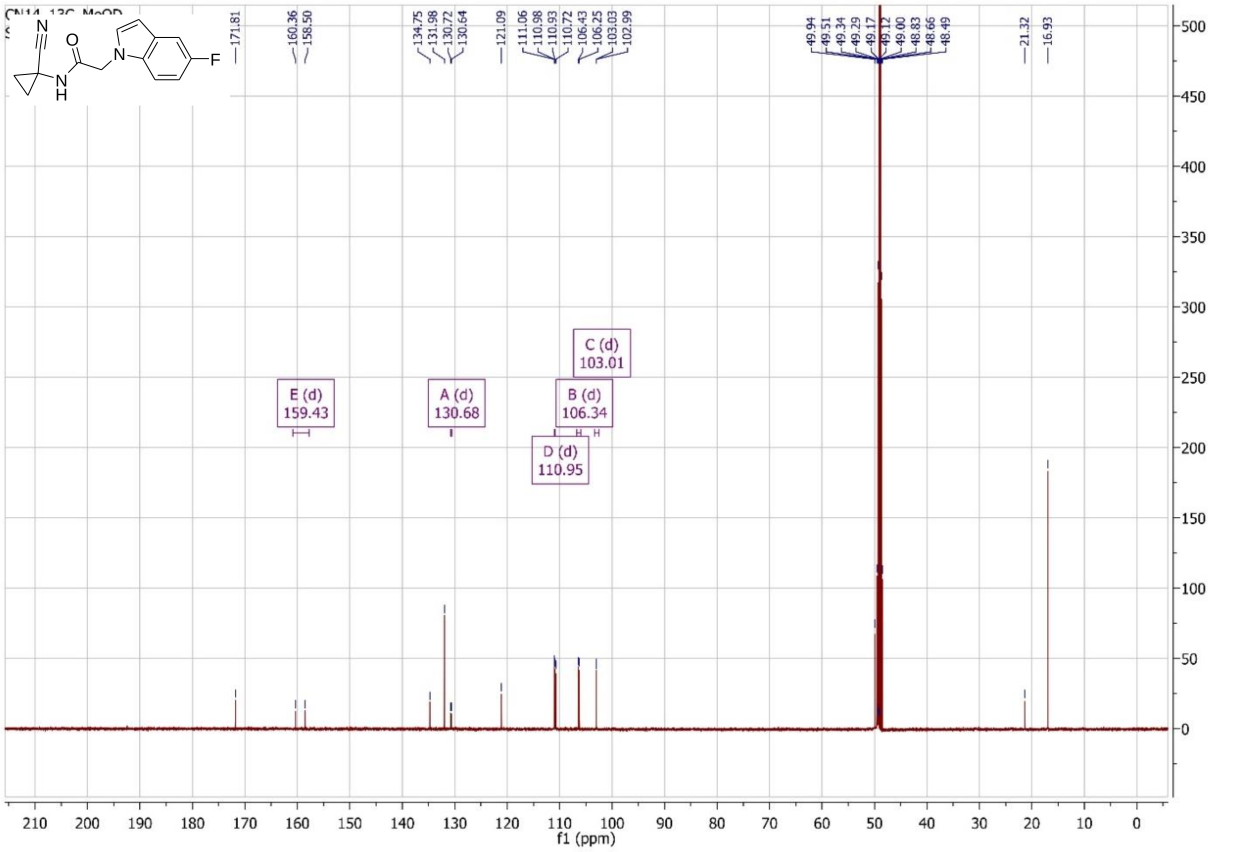

Figure S40. ^{13}C NMR spectrum of compound **8a**.

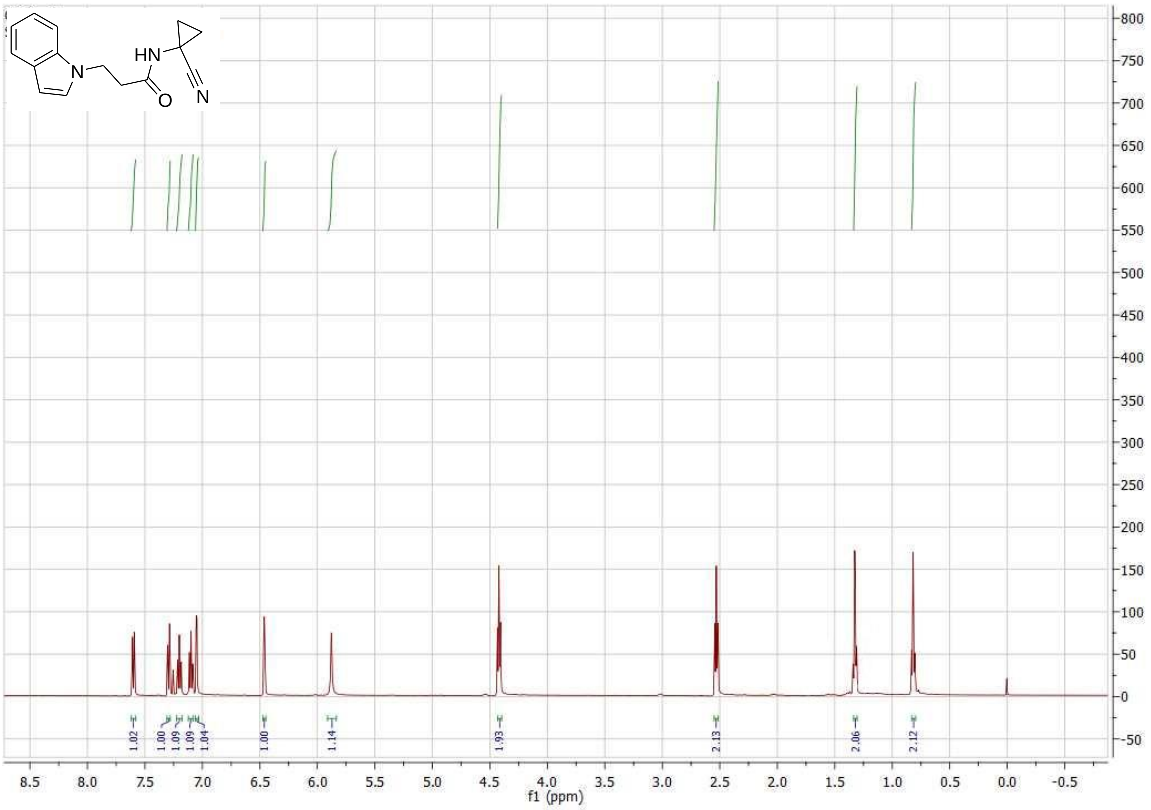

Figure S41. ^{1}H NMR spectrum of compound **5b**.

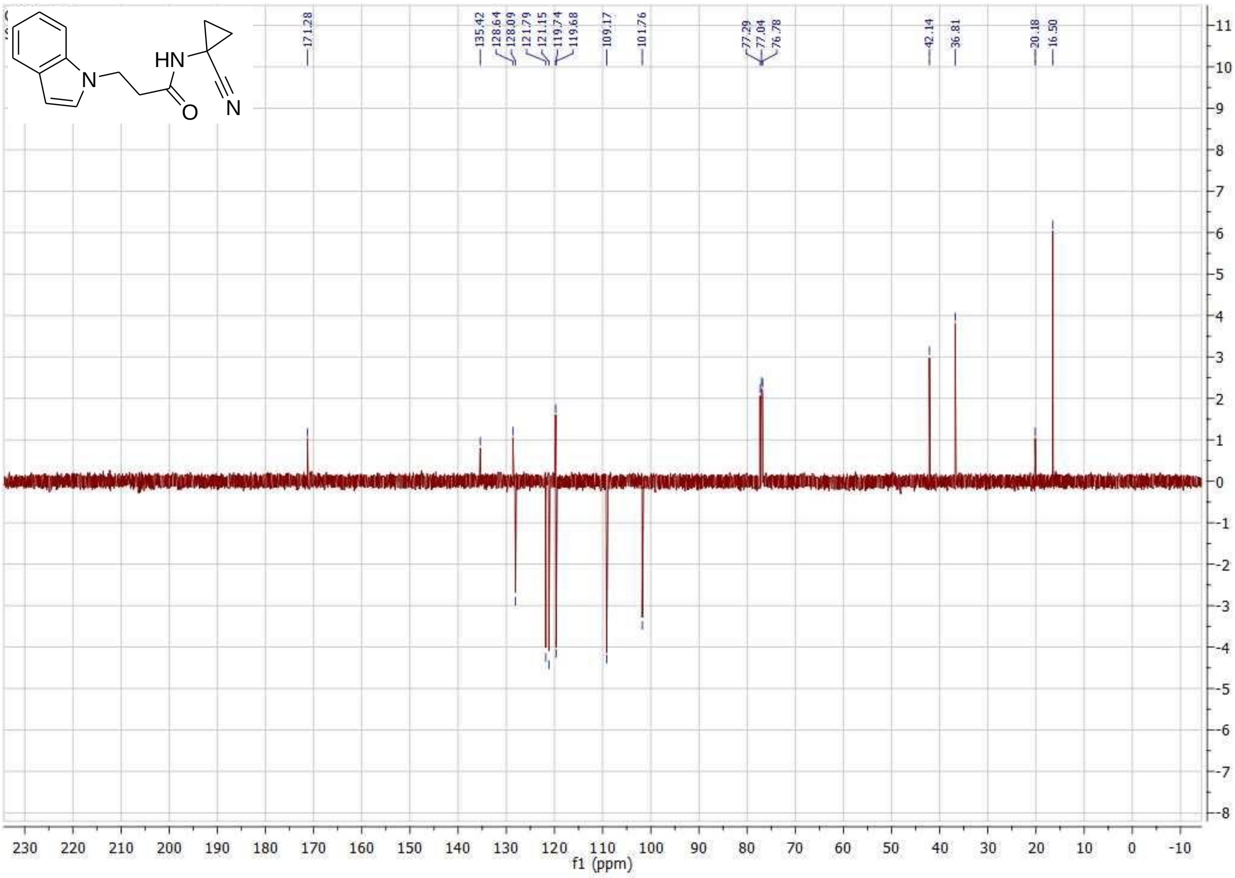

Figure S42. ^{13}C NMR spectrum of compound **5b.**

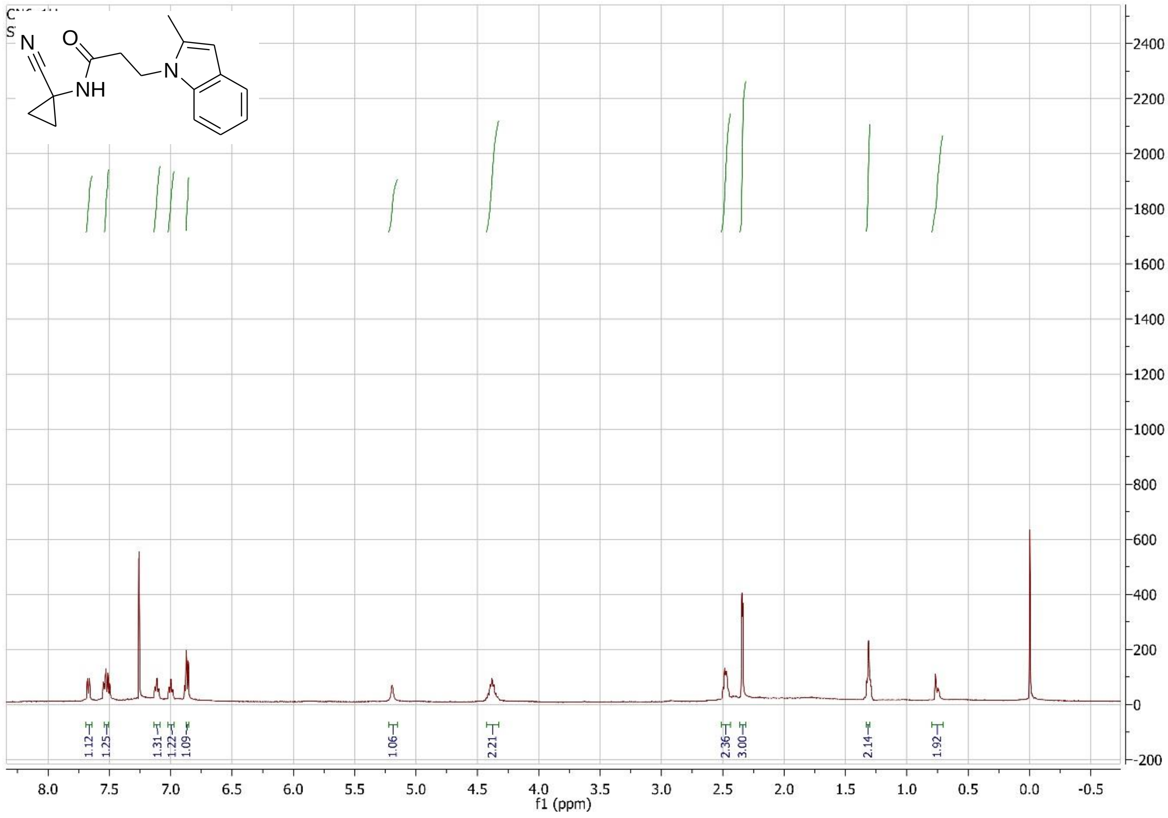

Figure S43. ^{1}H NMR spectrum of compound **6b**.

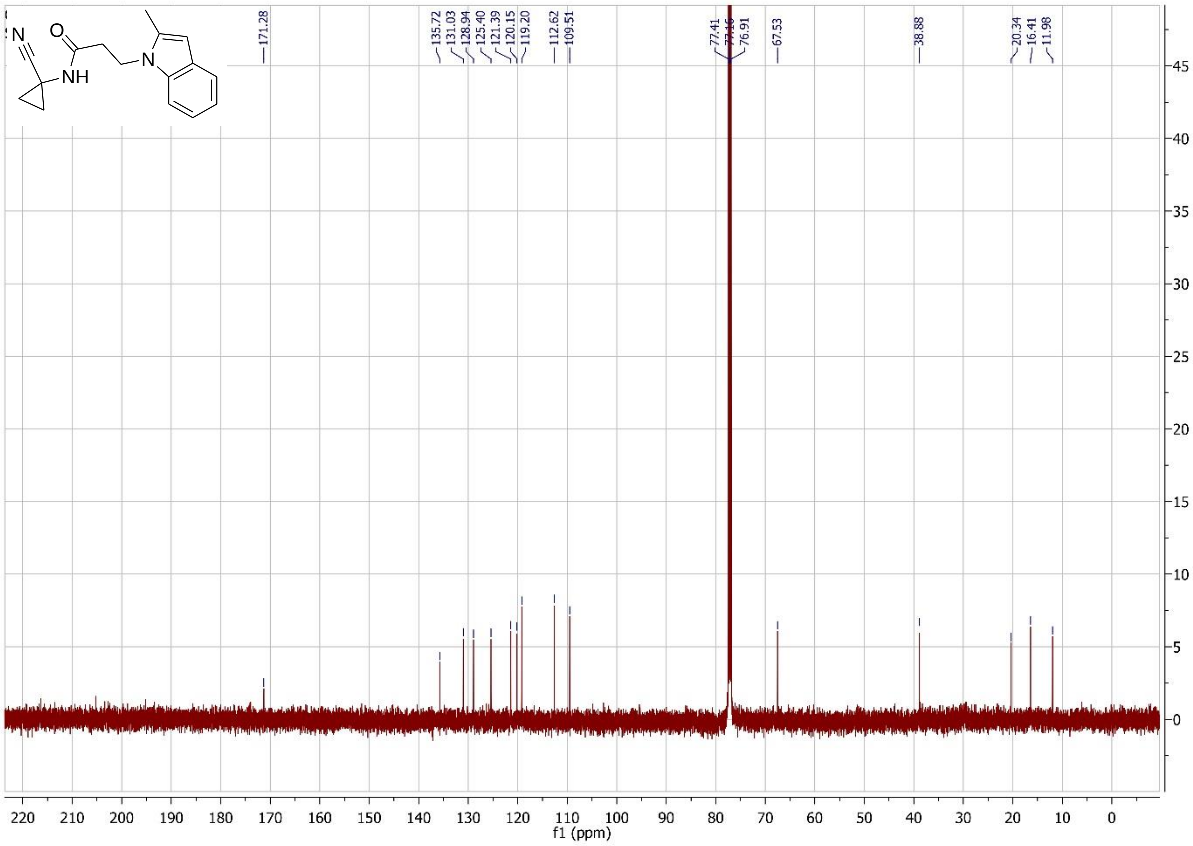

Figure S44. ^{13}C NMR spectrum of compound **6b**.

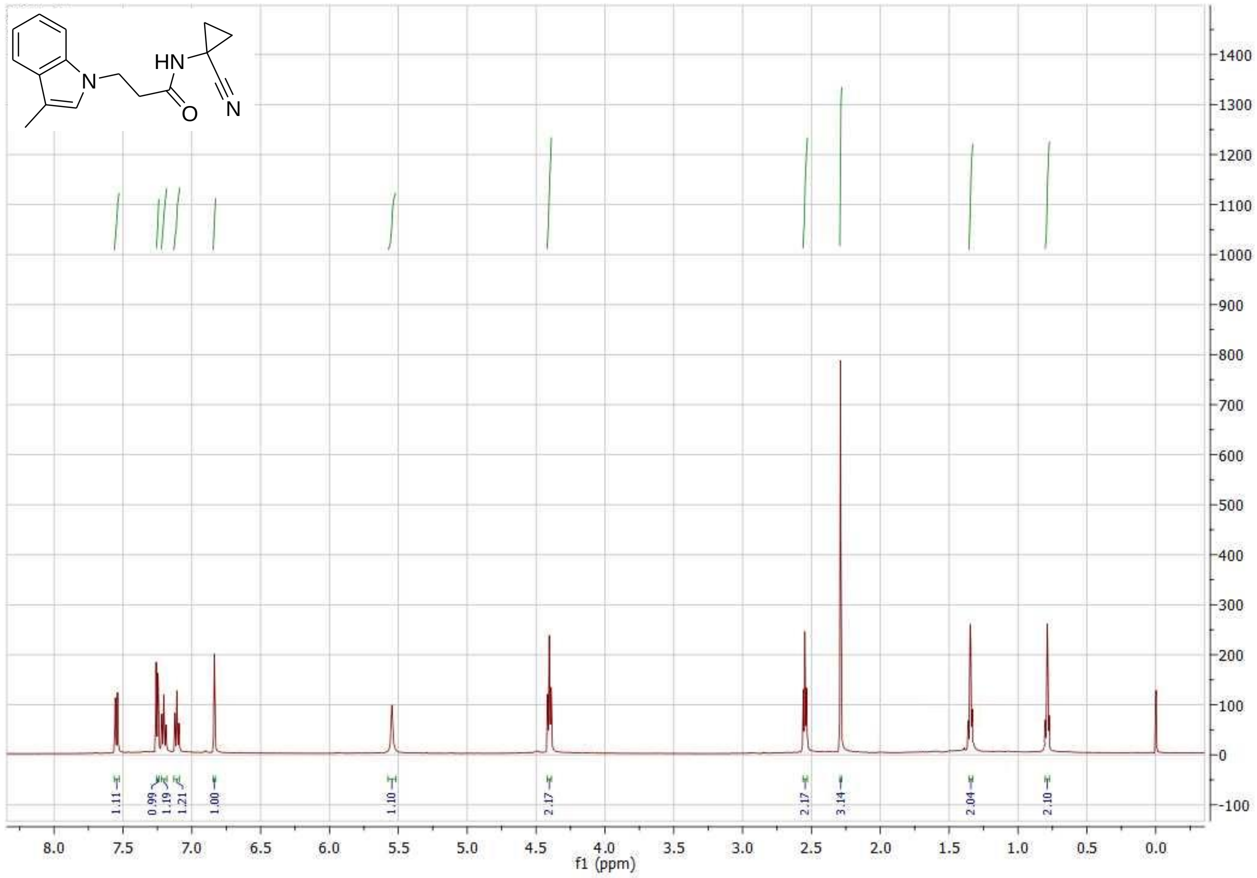

Figure S45. ^{1}H NMR spectrum of compound **7b.**

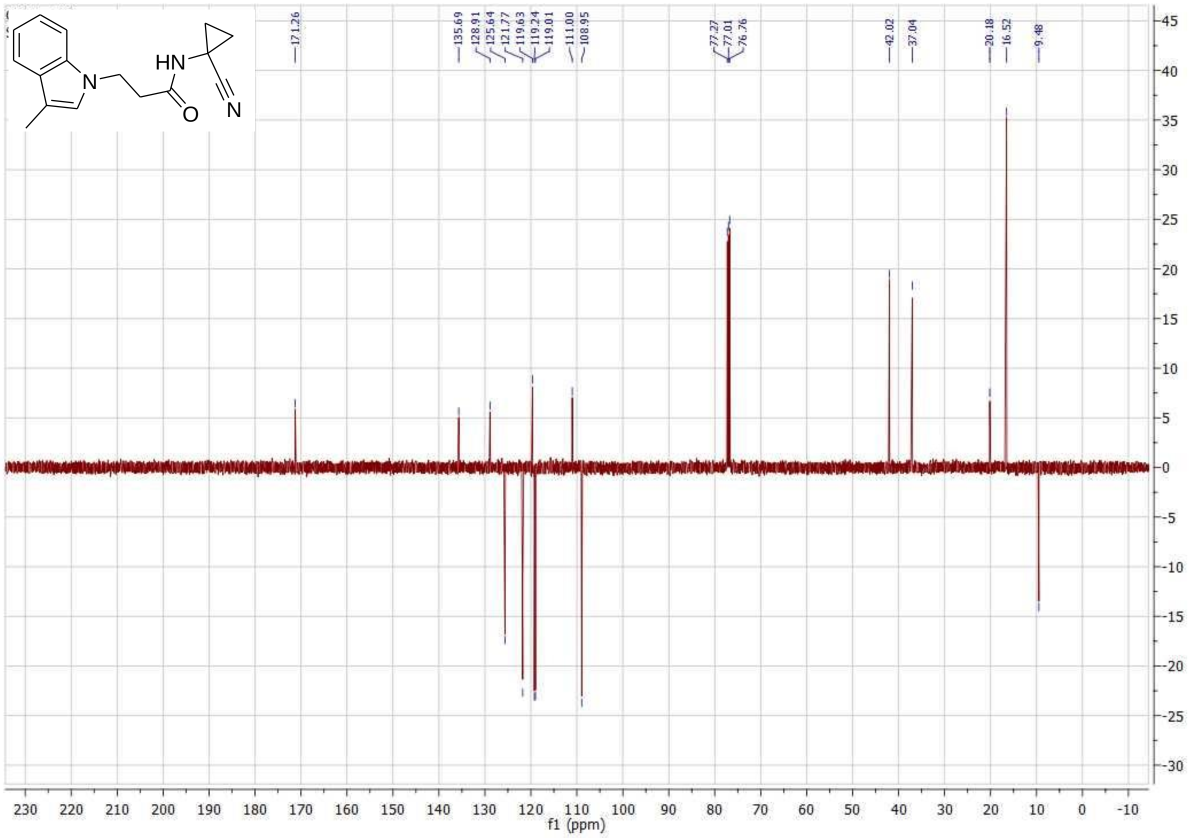

Figure S46. ^{13}C NMR spectrum of compound **7b**.

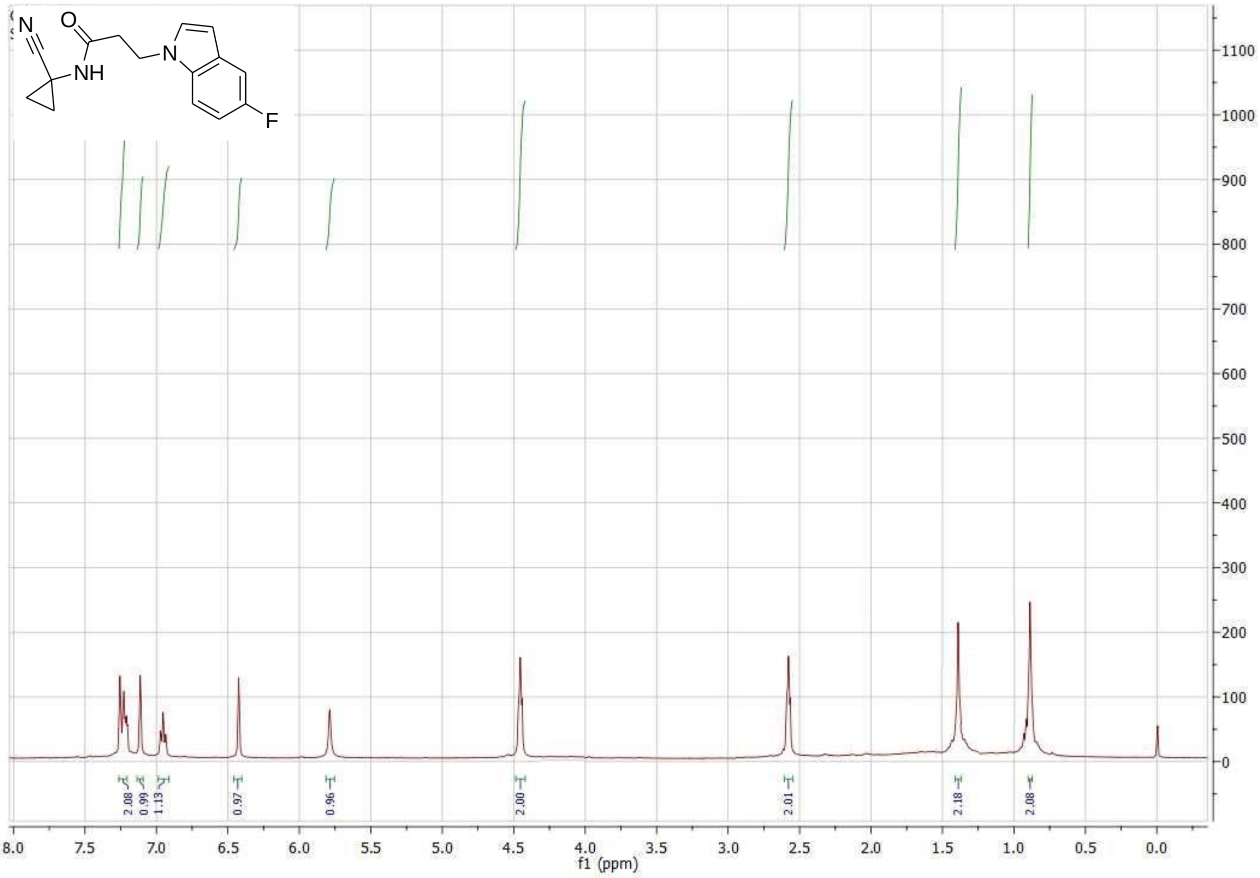

Figure S47. ^{1}H NMR spectrum of compound **8b.**

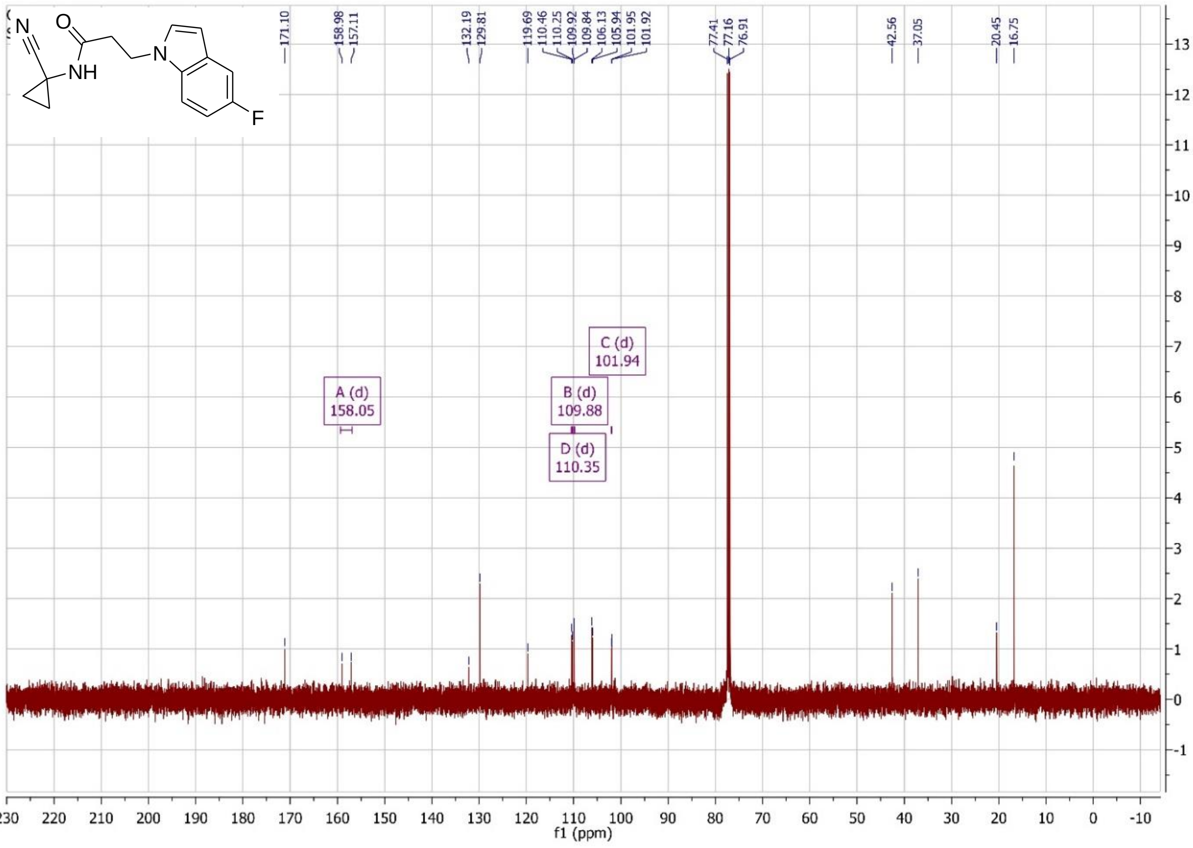

Figure S48. ^{13}C NMR spectrum of compound **8b**.
